# DNTTIP2 coordinates RNA exosome activities to ensure fidelity of human ribosome assembly

**DOI:** 10.1038/s41467-026-76536-x

**Published:** 2026-08-07

**Authors:** Agnese Pisano, Jessie Bourdeaux, Jarosław Mazur, Florine Roses, Yves Romeo, Elisabeth Petfalski, Tobias von Arx, Daniela Portugal-Calisto, Michaela Oborská-Oplová, Cohue Peña, Clément Chapat, David Tollervey, Anthony K. Henras, Vikram Govind Panse

**Affiliations:** 1 Institute of Medical Microbiology, University of Zurich, Zurich, Switzerland; 2 CBI, Université de Toulouse, CNRS, CBI, Toulouse, France; 3 Centre for Cell Biology, School of Biological Sciences, University of Edinburgh, Edinburgh, UK; 4 Faculty of Science, University of Zurich, Zurich, Switzerland

**Keywords:** RNA, RNA decay

## Abstract

The RNA exosome-associated helicase Mtr4/MTR4 (yeast/human) is recruited by adaptor proteins bearing Arch-Interacting Motifs (AIMs) to selectively degrade RNA substrates. Although the exosome targets diverse RNAs, only a few adaptors have been identified. Here, we extend the inventory of human adaptors to include a pre-tRNA splicing-ligase complex component, a spliceosome-associated factor, and DNTTIP2, a constituent of the small ribosomal subunit (40S) precursor, the 90S pre-ribosome. Structure-guided studies reveal how the DNTTIP2^AIM^-docked processive exosome core and its associated distributive exonuclease EXOSC10, which contact distant sites on the 90S pre-ribosome, cooperate to degrade part of the 5′-external transcribed spacer (5’-ETS), a key RNA scaffold that coordinates early 40S assembly. By contrast, productive pre-ribosomal RNA trimming within the 90S pre-ribosome necessitates EXOSC10, which safeguards against uncontrolled processive degradation by the DNTTIP2^AIM^-docked exosome core. We propose that multivalent contacts provide a mechanistic framework by which the RNA exosome coordinates its distinct enzymatic activities, ensuring selective processing and surveillance during ribonucleoprotein particle maturation.

## Abstract (web summary)

Here the authors identify human adaptors of the RNA exosome bound helicase MTR4, linking RNA surveillance to tRNA splicing, pre-mRNA splicing, and ribosome assembly. They show how DNTTIP2 directs processive and distributive activities of the RNA exosome to ensure fidelity of human ribosome assembly.

## Introduction

The selective processing and/or degradation of nascent RNAs is essential to maintain the fidelity of the transcriptome and regulate gene expression. Errors in RNA maturation or the accumulation of aberrant, cryptic, or unstable RNA transcripts can disrupt cellular homeostasis^1^. In eukaryotes, the RNA exosome, a conserved multi-subunit 3′−5′ exoribonuclease complex, plays a central role in RNA surveillance and turnover^2–4^. It degrades and/or processes a broad array of coding and non-coding RNAs, including precursor ribosomal RNAs (pre-rRNAs)^5^, small nuclear and nucleolar RNAs (snRNAs and snoRNAs)^6,7^, cryptic unstable transcripts (CUTs)^8^, promoter upstream transcripts (PROMPTs)^9^, and other non-productive transcripts produced by RNA polymerase I, II, and III^10,11^. Yet the mechanisms that selectively regulate in vivo RNA levels and quality remain incompletely understood.

The nine-subunit core of the eukaryotic RNA exosome (Exo-9) consists of six RNase PH-like subunits (Rrp41, Rrp42, Rrp43, Rrp45, Rrp46, and Mtr3) that form a barrel-shaped ring, and three cap proteins (Rrp4, Rrp40, and Csl4) with KH and S1 RNA-binding domains. Exo-9 is catalytically inert but serves as a scaffold to position two ribonucleases: Rrp6/EXOSC10 (yeast/human), a distributive 3′−5′ exonuclease, and Rrp44/DIS3 (yeast/human), a processive exonuclease which also harbours endonuclease activity^12–15^. Rrp6/EXOSC10 associates near the top of the barrel, where it engages the 3’-end of RNA for distributive processing. On the other hand, Rrp44/DIS3 is anchored at the base to engage with the 3’-end of RNA as it is threaded through the central channel toward its catalytic site for processive degradation^1,16^.

Specific RNA substrates must be selected for degradation versus processing. This process relies on different adaptor proteins that recognize specific RNA and protein features within ribonucleoprotein particles and then recruit the RNA exosome^1,16^. A central adaptor-interacting factor is the conserved Ski2-like RNA helicase Mtr4/MTR4 (yeast/human), which operates as a molecular bridge between RNA-bound adaptor proteins and the RNA exosome^1^. In addition to its helicase core, Mtr4/MTR4 contains a characteristic “Arch domain”, a large α-helical insertion that extends from the helicase core and engages with adaptor proteins to mediate specific RNA processing and surveillance events^17–20^. For example, in yeast, the large (60S) and small (40S) ribosomal subunit assembly factors Nop53 and Utp18 recruit the Mtr4-bound RNA exosome to trim 7S pre-rRNA to mature 5.8S rRNA and degrade part of the 5′-external transcribed spacer (5′-ETS) RNA scaffold, respectively^21–23^. This recruitment requires a short peptide sequence known as the Arch-interacting motif (AIM; consensus: LFXΦD, where X is any residue and Φ is hydrophobic)^23^. Structural studies have provided the molecular basis of how the AIM β-strand docks onto the KOW subdomain of the Mtr4-Arch to provide adaptor specificity^24–26^. Beyond canonical AIMs, alternative AIM variants have been identified. These include cysteine- and tryptophan-centred sequences (C-AIM and W-AIM), and a divergent isoleucine-centred motif (I-AIM: IEEF), which retains binding to MTR4-Arch but deviates from the consensus motif, suggesting a broader recognition capacity^25^.

In multicellular eukaryotes, the complexity of the transcriptome is marked by pervasive transcription, non-coding RNA diversity, and alternative processing pathways, suggesting a greater need for surveillance by the RNA exosome. Yet relatively few MTR4 Arch-interacting adaptors have been identified^1^. These include the Poly(A) Exosome Targeting (PAXT) complex, containing ZFC3H1 that recruits the exosome to polyadenylated long noncoding RNAs, such as enhancer RNAs (eRNAs), upstream antisense RNAs (uaRNAs), and prematurely terminated RNAs^27^. Another adaptor, ZCCHC8, is present within the Nuclear Exosome Targeting (NEXT) complex that targets PROMPTs and histone mRNAs for degradation^25,28–30^. A third adaptor, NRDE2, binds to the MTR4-Arch via a canonical AIM and an additional RecA2-interacting segment, locking MTR4 into a closed conformation and preventing engagement with the polyadenylating TRAMP complex ^26^. In addition to these transcript-surveillance pathways, MTR4 Arch-interacting adaptors have also been implicated in pre-rRNA processing, including human ribosome assembly factors NOP53^23,24^ and NVL^25^. While NOP53 has been linked to the recruitment of MTR4-bound RNA exosome to trim 12S pre-rRNA to 5.8S rRNA during 60S subunit assembly, the precise contribution of NVL to the pre-rRNA processing pathway remains unclear.

Despite advances in sequencing technologies that have revealed the enormous diversity of eukaryotic RNAs, the repertoire of exosome adaptors that regulate their levels remains incomplete. In this study, we extend the inventory of adaptors that interact with MTR4 through an unbiased yeast two-hybrid (Y2H) screening approach. These adaptors connect the RNA exosome to different essential pathways critical for gene expression, including a metazoan adaptor that directs the degradation of a 5′-ETS RNA fragment that scaffolds early ribosome assembly. Structure-guided functional studies of this long-sought MTR4 adaptor illuminate how selective RNA processing and surveillance are enforced by the exosome during pre-ribosome maturation.

## Results

### A Y2H screen extends the inventory of MTR4 interactors

To expand the repertoire of human AIM sequences, we performed a Y2H screen using the Arch domain of MTR4 (MTR4-Arch) (Supplementary Fig. 1a, left panel) as bait against a prey library derived from human placenta cDNA. The screen recovered different fragments of known MTR4-Arch adaptors containing canonical AIMs and AIM variants (Supplementary Fig. 1a, right panel). These included the nucleolar localised 60S assembly factors NOP53 and the AAA^+^-ATPase NVL, as well as the C-AIM-containing fragment of ZCCHC8, a core component of the NEXT complex (Supplementary Fig. 1a, right panel; 1b). In addition to established adaptors, the screen identified fragments of CLE7, LAS2, CDC5L, and ZFC3H1 that lack a recognizable AIM or its variants but still showed robust interaction with MTR4-Arch (Supplementary Fig. 1a, right panel; 1c). Among these, CLE7 (also known as RTRAF or CGI-99) is a non-catalytic subunit of the hetero-pentameric tRNA splicing ligase complex (tRNA-LC)^31^, which ligates 5′ and 3′ tRNA halves after intron excision by the TSEN/CLP1 complex^32^. Y2H studies revealed that a C-terminal fragment of CLE7 (residues 206-244; CLE7^206–244^) interacts with the KOW subdomain of MTR4-Arch (Supplementary Fig. 2a, upper panel, rows 1–2), and this interaction was confirmed by performing in vitro binding assays (Supplementary Fig. 2a, lower panel, lanes 5–6). Structural studies of the yeast Mtr4:Nop53^AIM^ complex have shown that two conserved arginines within the KOW subdomain form salt bridges with a conserved aspartate in the AIM, a feature of canonical Mtr4-Arch:AIM binding^24^. We found that the CLE7^206–244^ interaction with MTR4-Arch requires the corresponding R658 and R743 within the KOW subdomain, as judged by Y2H (Supplementary Fig. 2a, upper panel, rows 3–4) and in vitro binding assays (Supplementary Fig. 2a, lower panel, lanes 7–8), despite lacking an identifiable AIM or its variant.

Genome-wide association studies have identified the loss of the nuclear localised protein LAS2 as a key step in tumorigenesis, although its precise role in preventing 40% of lung adenocarcinomas remains unexplored^33^. CDC5L, in turn, is a core component of the conserved PRP19 complex, which functions in multiple biological processes, including the catalytic activation of the spliceosome during pre-mRNA splicing^34,35^. Similar interaction profiles of MTR4-Arch with LAS2^487-533^ and CDC5L^579-743^ fragments were observed in Y2H studies (Supplementary Fig. 2b, upper panel, rows 1–4; Fig. 2c, rows 1–4) and in vitro binding assays (Supplementary Fig. 2b, lower panel, lanes 5–8). All these findings show that CLE7, LAS2, and CDC5L bind to MTR4-Arch.

To gain insights into the structural basis of these interactions, we modelled MTR4-Arch:adaptor fragment complexes using AlphaFold2. High-confidence models were obtained for MTR4 complexes with NOP53^1–201^, ZCCHC8^150–191^, and NVL^1–370^ (Supplementary Fig. 3a–c and Supplementary Data 1–3), all of which contain canonical or known AIMs. AlphaFold2 failed to reliably model MTR4-Arch complexes with CLE7^206–244^, LAS2^487–533^, and CDC5L^579–743^ (Supplementary Fig. 4a–c and Supplementary Data 4–6), reflecting a divergent binding mode. Nevertheless, Y2H and biochemical studies (Supplementary Fig. 2a–c) indicated that binding relies on conserved basic residues of MTR4-Arch, suggesting mechanistic parallels with canonical AIM interactions.

The ZFC3H1^1139–1321^ fragment interacts with MTR4-Arch in a distinct manner. Y2H analysis showed that neither the KOW subdomain nor the conserved arginines R658 and R743 within MTR4-Arch were required for ZFC3H1^1139–1321^ binding (Supplementary Fig. 2d). AlphaFold2 modelling of the MTR4-Arch:ZFC3H1^1139–1321^ complex resulted in a high-confidence structure (Supplementary Figs. 2e, 3d and Supplementary Data 7), revealing an interface that does not resemble a canonical interaction. Instead, the model shared features with a complex formed between fission yeast Mtr4 (Mtl1) and the zinc-finger protein Red1 (human ZFC3H1) within the MTREC complex, an 11-subunit nuclear-localised RNA degradation machinery (Supplementary Fig. 2e)^36,37^. We conclude that our screen extends the inventory of MTR4-Arch interacting factors.

### A metazoan-specific segment within DNTTIP2 harbours a canonical AIM

**Fig. 1:**
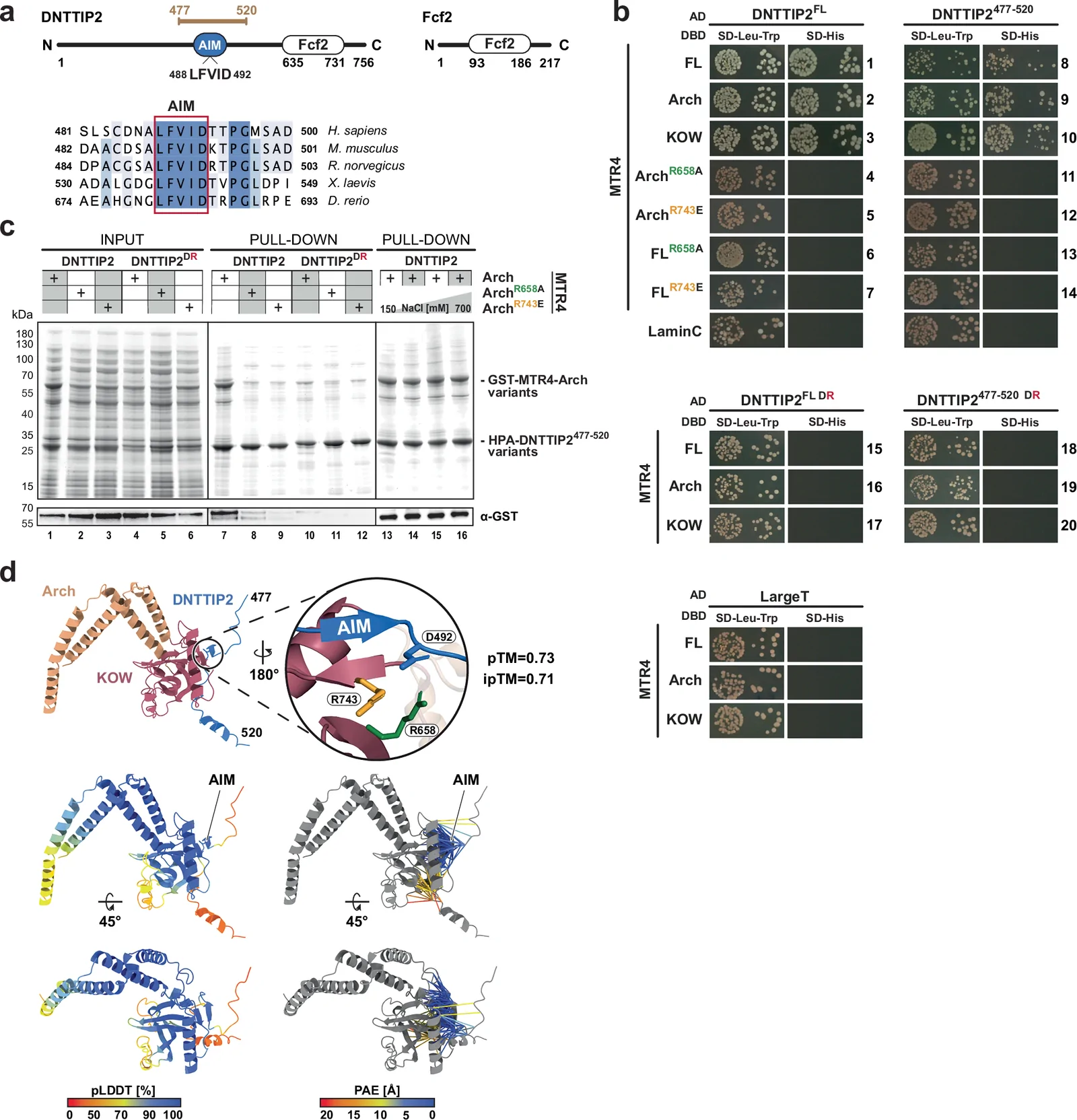
A metazoan-specific segment within DNTTIP2 contains a canonical AIM. **a** Domain representation of human DNTTIP2 (left) and its yeast homolog Fcf2 (right). The AIM of DNTTIP2 is coloured in blue. The peptide used in Y2H screen and for sequence alignment is marked in brown. Lower panel: sequence alignment of DNTTIP2 from higher eukaryotes with the AIM framed in red. **b** Y2H analysis testing interactions of MTR4 variants with full-length DNTTIP2 (DNTTIP2^FL^), DNTTIP2^477-520^ fragment, and the D492R (DR) substitution. Plasmids encoding the tested proteins fused to the LexA DNA-binding domain (DBD) and GAL4 activation domain (AD), as indicated, were transformed into the NMY32 strain. Transformants were spotted on selective SD-Leu-Trp and SD-His media and incubated at 30 °C for 3-4 days. LaminC and LargeT were used as negative controls. **c** GST-tagged variants of MTR4 were co-expressed with His_6_-ProteinA (HPA)-DNTTIP2^477-520^ and its AIM mutant (DR). In vitro binding assays were performed using a Ni-NTA resin in a buffer containing increasing NaCl concentrations (150–700 mM). The bound proteins were analysed by SDS-PAGE and Western blotting using α-GST antibodies. **d** AlphaFold2 generated model of the MTR4-Arch domain (residues 579-842) and DNTTIP2^477-520^ fragment. The inset highlights the interaction between two arginine residues (R658 in green and R743 in yellow) of MTR4 and an invariant aspartate residue (D492 in blue) of DNTTIP2. Prediction confidence was assessed using the predicted distance difference test (pLDDT), predicted aligned error (PAE), and pTM/ipTM scores. Pseudo-bonds reflecting the PAE between residue pairs were displayed for distances up to 8 Å. The AlphaFold2 predicted structure as a PDB-format coordinate file is provided as Supplementary Data 8. Source data are provided as a Source Data file.

Among the MTR4-Arch interactors identified in the screen, we focused on a fragment encompassing residues 477–520 of the nucleolar localised ribosome assembly factor DNTTIP2 (DNTTIP2^477–520^) (Supplementary Fig. 1a, right panel). The C-terminal region of DNTTIP2 (residues 635–731) is homologous to yeast Fcf2 (residues 93–186), an essential component of the earliest 40S subunit precursor, the 90S pre-ribosome (Fig. 1a). Cryo-EM reconstructions showed that this region within DNTTIP2, like Fcf2, binds to UTP6, a subunit of the UTP-B complex that is co-transcriptionally recruited to the scaffolding 5′-ETS RNA during 90S pre-ribosome assembly^38^. In comparison to unicellular eukaryotes, metazoan DNTTIP2 homologs exhibit a significantly longer ∼600 residue N-terminal extension (Fig. 1a and Supplementary Fig. 5a), suggesting that this region may have acquired additional functions. Close inspection of the DNTTIP2^477–520^ fragment identified in the Y2H screen revealed a canonical AIM that is conserved only in metazoans (LFVID; residues 488–492) (Fig. 1a). Y2H assays showed that full-length DNTTIP2 and the DNTTIP2^477–520^ fragment robustly interact with the KOW subdomain of MTR4-Arch (Fig. 1b, rows 1–3 and 8–10). Biochemical studies showed that DNTTIP2^477–520^ fragment forms a robust salt-stable complex with GST-tagged MTR4-Arch (Fig. 1c, lane 7) that remained intact up to at least 700 mM NaCl (Fig. 1c, lanes 13–16). Structure predictions using AlphaFold2 indicated canonical salt bridges formed between the acidic residue (D492) within the LFVID sequence of DNTTIP2^477–520^ fragment and two basic residues (R658 and R743) within the MTR4 KOW subdomain (Fig. 1d, inset; Supplementary Data 8). Substitution of R658 or R743 in MTR4 (R658A, R743E) disrupted binding to full-length DNTTIP2 and the DNTTIP2^477–520^ fragment as judged by Y2H and in vitro binding assays (Fig. 1b, rows 4–7 and 11-14; Fig. 1c, lanes 7–9). Similarly, substitution of the conserved D492 in DNTTIP2^477–520^ to arginine (DNTTIP2^DR^) abolished interactions with MTR4 or MTR4-Arch (Fig. 1b, rows 15–17 and 18–20; Fig. 1c, lane 10). These results indicate that the MTR4-Arch:DNTTIP2^477–520^ complex is assembled via a canonical interaction. We term this conserved LFVID sequence as DNTTIP2^AIM^.

### A role for DNTTIP2^AIM^ in human 5′-ETS RNA degradation

**Fig. 2:**
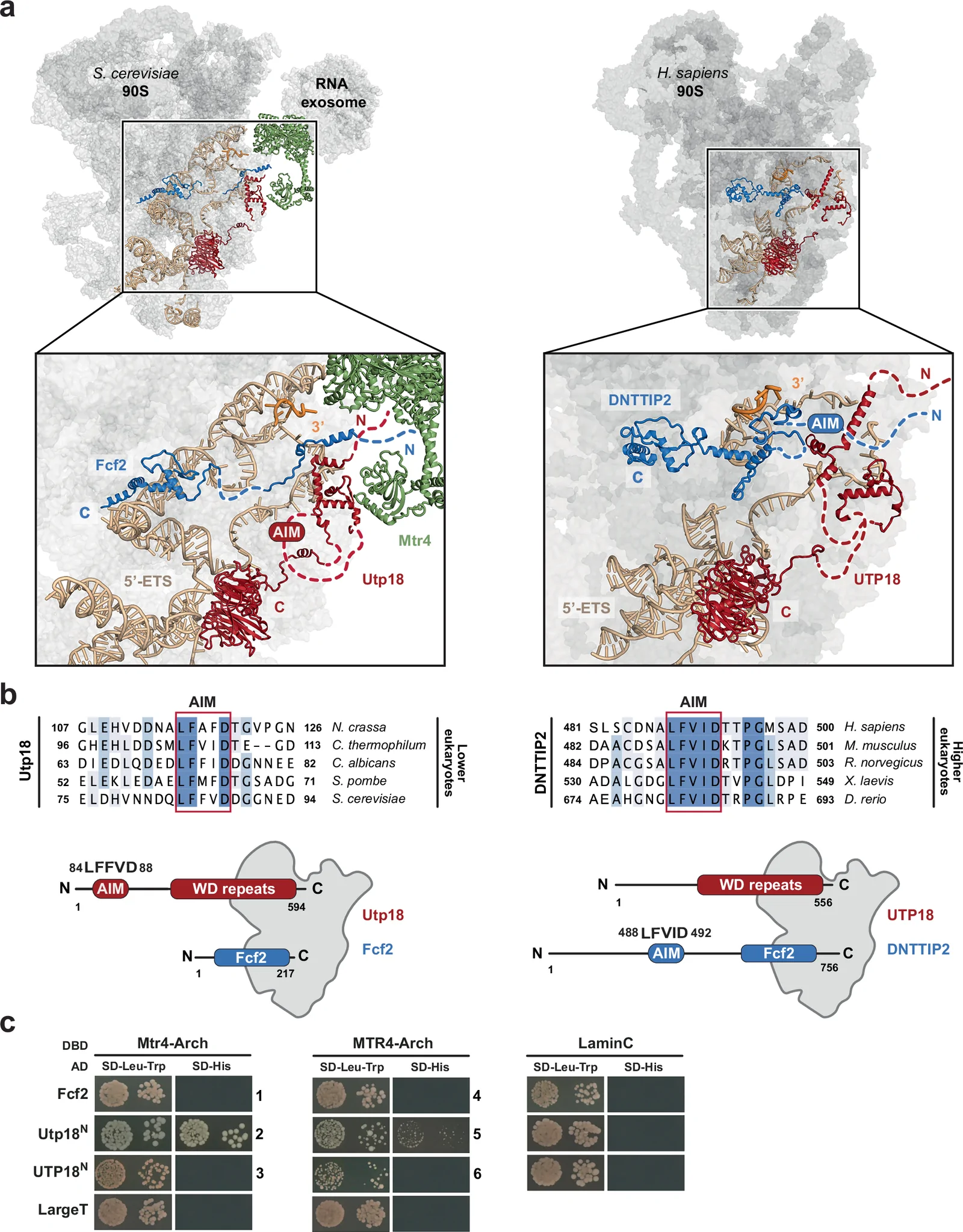
Targeting of the RNA exosome to the 5′-ETS RNA in yeast and humans. **a** Left: cryo-EM structure of the yeast 90S pre-ribosome together with the RNA exosome (PDB: 7AJT), with a detailed view of the 5’-ETS region. Fcf2 (blue), Utp18 (red), Mtr4 (green), 5’-ETS (beige), and the 5’-ETS 3’ end (orange) are indicated. Right: cryo-EM structure of the human 90S pre-ribosome (PDB: 7MQ8), with a detailed view of the 5’-ETS regions. DNTTIP2 (blue), UTP18 (red), 5’-ETS (beige), and the 5’-ETS 3’ end (orange) are indicated. **b** Top left: Sequence alignment of the Utp18 AIM-containing sequence across lower eukaryotes. Lower left: domain organization of AIM-containing Utp18 and Fcf2 in yeast. Top right: Sequence alignment of the DNTTIP2/Fcf2 across higher eukaryotes. Lower right: domain organization of human AIM-lacking UTP18 and AIM-containing DNTTIP2. **c** Y2H analysis testing interactions of yeast Mtr4-Arch and human MTR4-Arch domains with Fcf2, and yeast and human Utp18/UTP18 N-terminus. Plasmids encoding the tested proteins fused to the LexA DNA-binding domain (DBD) and GAL4 activation domain (AD), as indicated, were transformed into the NMY32 strain. Transformants were spotted on selective SD-Leu-Trp and SD-His media and incubated at 30 °C for 3–4 days. LaminC and LargeT were used as negative controls.

In yeast, the RNA exosome is recruited to the 90S pre-ribosome by the assembly factor Utp18, which contains a canonical AIM (Utp18^AIM^). This interaction ensures that part of the 5′-ETS RNA is degraded in a timely manner, presumably during late nucleolar maturation. Cryo-EM studies^39^ showed that the N-terminal region of Utp18 is positioned near the 3′-end of the 5′-ETS RNA (Fig. 2a, left panel, inset). An unresolved segment of this region contains a canonical AIM (Utp18^AIM^, Fig. 2b, left panel), consistent with a role in guiding the Mtr4-associated RNA exosome toward its substrate. A similar architectural organization was observed in the human 90S pre-ribosome^38^, wherein the N-terminal region of UTP18 (UTP18^N^) is in the vicinity of the 3′-end of the 5′-ETS RNA (Fig. 2a, right panel, inset). Yet, sequence analysis of UTP18^N^ revealed no AIM or its variants (Supplementary Fig. 5b), a finding further supported by Y2H assays that showed no interaction between UTP18^N^ and MTR4-Arch (Fig. 2c, row 6). In contrast, a robust interaction was observed between yeast Utp18^N^ and Mtr4-Arch, but not MTR4-Arch (Fig. 2c, rows 2 and 5). These results do not support a role for UTP18 in recruiting the MTR4:RNA exosome complex to the human 90S pre-ribosome.

**Fig. 3:**
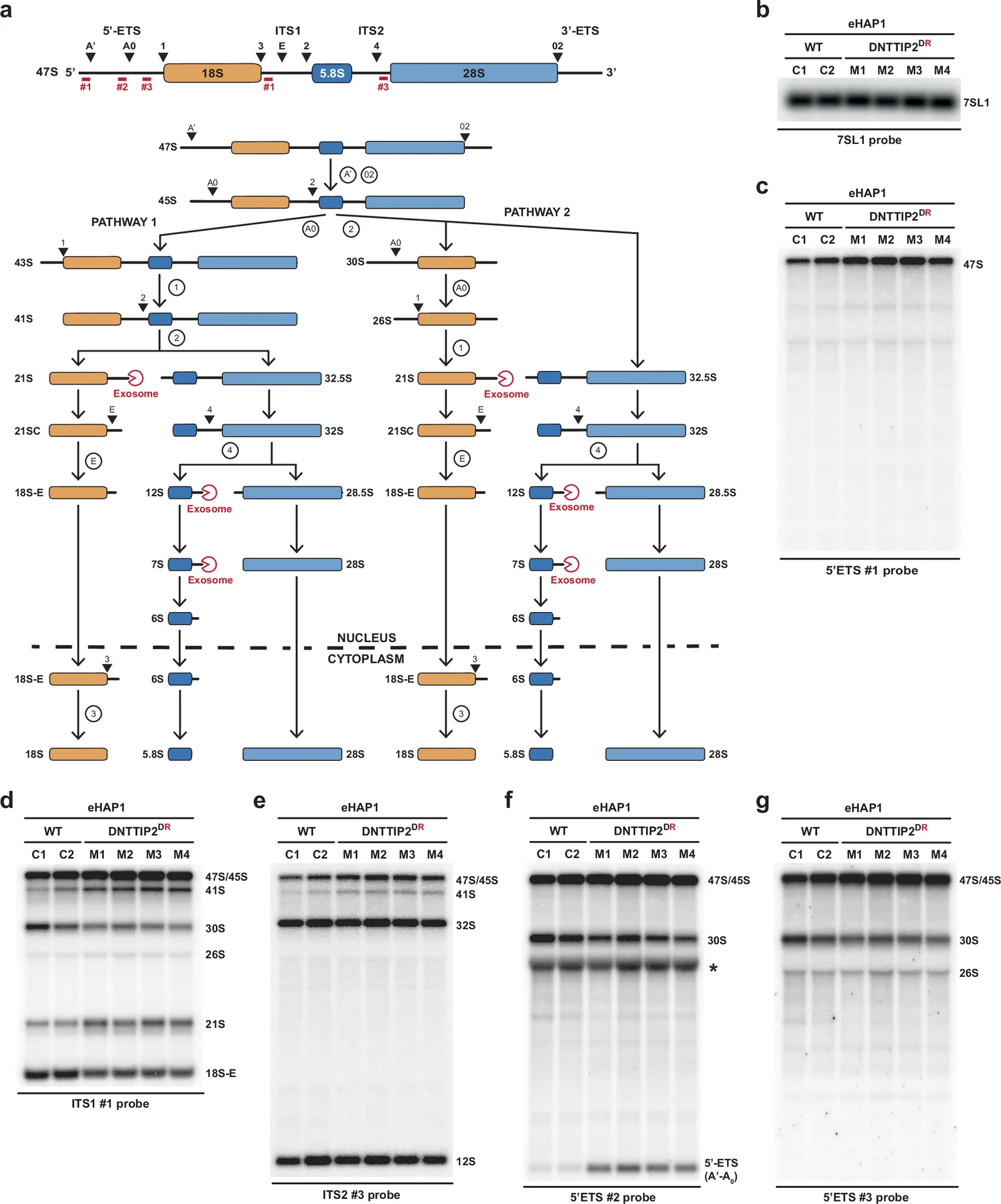
5’-ETS A’-A0 RNA fragment degradation requires DNTTIP2^AIM^. **a** Schematic overview of human pre-rRNA processing pathways. Pathway 1 proceeds via cleavage of the 45S precursor at sites A0 and 1, generating the 41S product. This species is subsequently cleaved at site 2, producing the 21S and 32.5S intermediates that are then converted to the mature 18S and 5.8S/28S rRNAs, respectively. Pathway 2 involves cleavage of the 45S precursor at site 2, separating it into 30S and 32.5S intermediates. External (ETS) and internal (ITS) transcribed spacers are indicated. Positions of the 5’ETS #1, 5’ETS #2, 5’ETS #3, ITS1 #1, and ITS2 #3 probes used in Northern blotting experiments are indicated. **b**–**g** Two control eHAP1 cell lines expressing wild-type DNTTIP2 (C1-C2) and four independent mutant cell lines expressing the DNTTIP2^DR^ variant (M1-M4) were grown to mid-confluence. Total RNA extracted from these cells was analysed by Northern blotting using the following probes: **b** 7SL1 (loading control), **c** 5’ETS #1, **d** ITS1 #1, **e** ITS2 #3, f 5’ETS #2, and **g** 5’ETS #3. The rRNA precursors detected by the individual probes are indicated on the right. The asterisk marks a cross-hybridizing band corresponding to the mature 28S rRNA. The precise positions of the different probes on the 47S pre-rRNA are shown in Fig. 3a. Source data are provided as a Source Data file.

The DNTTIP2^AIM^ (Fig. 2b, right panel) lies within an unresolved region that also localizes near the 3′-end of the 5′-ETS RNA within the human 90S pre-ribosome (Fig. 2a, right panel, inset), implicating a conserved spatial mechanism for RNA exosome recruitment. We hypothesized that DNTTIP2 is the elusive adaptor that recruits the RNA exosome to the human 90S pre-ribosome to degrade 5′-ETS RNA. To investigate the role of DNTTIP2^AIM^ during human pre-rRNA processing (Fig. 3a), we employed CRISPR-Cas9 genome editing to mutate the conserved AIM motif in the eHAP1 cell line. Specifically, the conserved aspartate residue within DNTTIP2^AIM^ was replaced with arginine, a mutation that disrupts interaction with basic residues on the MTR4-Arch in vitro (Fig. 1b, c). Four independent DNTTIP2^DR^ mutant clones (M1–M4) were selected for analysis. Two control lines (C1, C2), which underwent genome editing but were not mutated at the DNTTIP2 locus, were included to exclude off-target effects. Western blotting analysis and quantification confirmed that the DNTTIP2^DR^ protein was expressed at levels comparable to wild type, indicating that the D to R substitution does not affect protein stability (Supplementary Fig. 6a). Metabolic (MTS) assays revealed a reduction in cell proliferation across the DNTTIP2^DR^-expressing lines over a four-day experiment (Supplementary Fig. 6b), correlating with a significant decrease in global protein synthesis. Using a surface sensing of translation (SUnSET) assay^40^, we detected an ~30% reduction in puromycin incorporation in DNTTIP2^DR^-expresssing cells as compared to wild-type DNTTIP2 control cells (Supplementary Fig. 6c). Sucrose gradient sedimentation analyses of lysates derived from DNTTIP2^DR^-expresssing cells showed a significant reduction in the levels of mature 40S subunits as compared to those derived from wild-type DNTTIP2-expressing cells (Supplementary Fig. 6d). Quantification of the 40S/60S peak ratio revealed a 2 to 4-fold decrease in the DNTTIP2^DR^-expresssing cells as compared to the control wild-type DNTTIP2-expressing clones (Supplementary Fig. 6d). All these data support the model that the DNTTIP2^AIM^ sustains protein synthesis and cell proliferation, due to its role in driving 40S subunit assembly.

To investigate the role of DNTTIP2^AIM^ during pre-rRNA processing total RNA was extracted from DNTTIP2^DR^ and control cell lines, separated by denaturing agarose gel electrophoresis, and subjected to Northern blotting using probes targeting 5′-ETS, ITS1, and ITS2 regions (Fig. 3a), to detect the different pre-rRNA processing intermediates (Fig. 3c–g: quantified in Supplementary Fig. 7). A probe that base pairs with 7SL1 RNA, the scaffold of the signal recognition particle (SRP), was used as a loading control and for normalization of the Northern blotting data (Fig. 3b). Hybridization with the 5′-ETS #1 probe revealed an increase of the 47S pre-rRNA levels in DNTTIP2^DR^ lines, indicating a delay in cleavage at site A′ (Fig. 3c and Supplementary Fig. 7a). This conclusion is supported by the 30% reduction in 30S pre-rRNA levels, detected with probe ITS1 #1 (Fig. 3d and Supplementary Fig. 7b), consistent with the known requirement for MTR4 in cleavage at the A′ site^41^. In addition, DNTTIP2^DR^ cells displayed an increase in 41S pre-rRNA levels (Fig. 3d, e and Supplementary Fig. 7b), showing that this intermediate is produced efficiently, and that cleavages at sites A0 and A1 are less affected relative to cleavage at site A’. The levels of the downstream 21S pre-rRNA intermediate were increased by nearly 30%, correlating with a reduction of the same magnitude in production of the subsequent 18S-E pre-rRNA (Fig. 3d and Supplementary Fig. 7b), supporting a role of MTR4 in 3′ end trimming of this species^42^. These results indicate that efficient 47S pre-rRNA processing to 18S-E pre-rRNA requires intact MTR4-Arch:DNTTIP2^AIM^ interactions.

To assess 5′-ETS RNA levels, Northern blotting with probe 5′ETS #2 revealed a robust, 3-fold increase in the accumulation of the A′-A0 fragment in DNTTIP2^DR^ cells (Fig. 3f and Supplementary Fig. 7c). This finding is consistent with previous reports showing that MTR4 depletion blocks degradation of this fragment^41,43,44^. In contrast, the 5’-ETS RNA species mapping upstream (5’ end of the 47S precursor to site A’) and downstream (A0-1 fragment) of the A′-A0 fragment did not accumulate (Fig. 3c, g), suggesting that their turnover proceeds independently of MTR4:DNTTIP2^AIM^ interactions, and relies solely on XRN2 5′−3′ exonucleolytic activity^41^. No significant differences were observed in the levels of 32S and 12S precursors to the mature 28S and 5.8S rRNAs within the 60S subunit using probe ITS2 #3 between mutant and control cells (Fig. 3e and Supplementary Fig. 7d), indicating that DNTTIP2^AIM^ is dispensable for pre-rRNA processing along the 60S subunit assembly pathway. We conclude that the recruitment of MTR4-associated RNA exosome via DNTTIP2^AIM^ is required for efficient degradation of the A′-A0 fragment within 5′-ETS RNA.

### A role for Rrp6 C-terminal tail in 5’-ETS RNA degradation

**Fig. 4:**
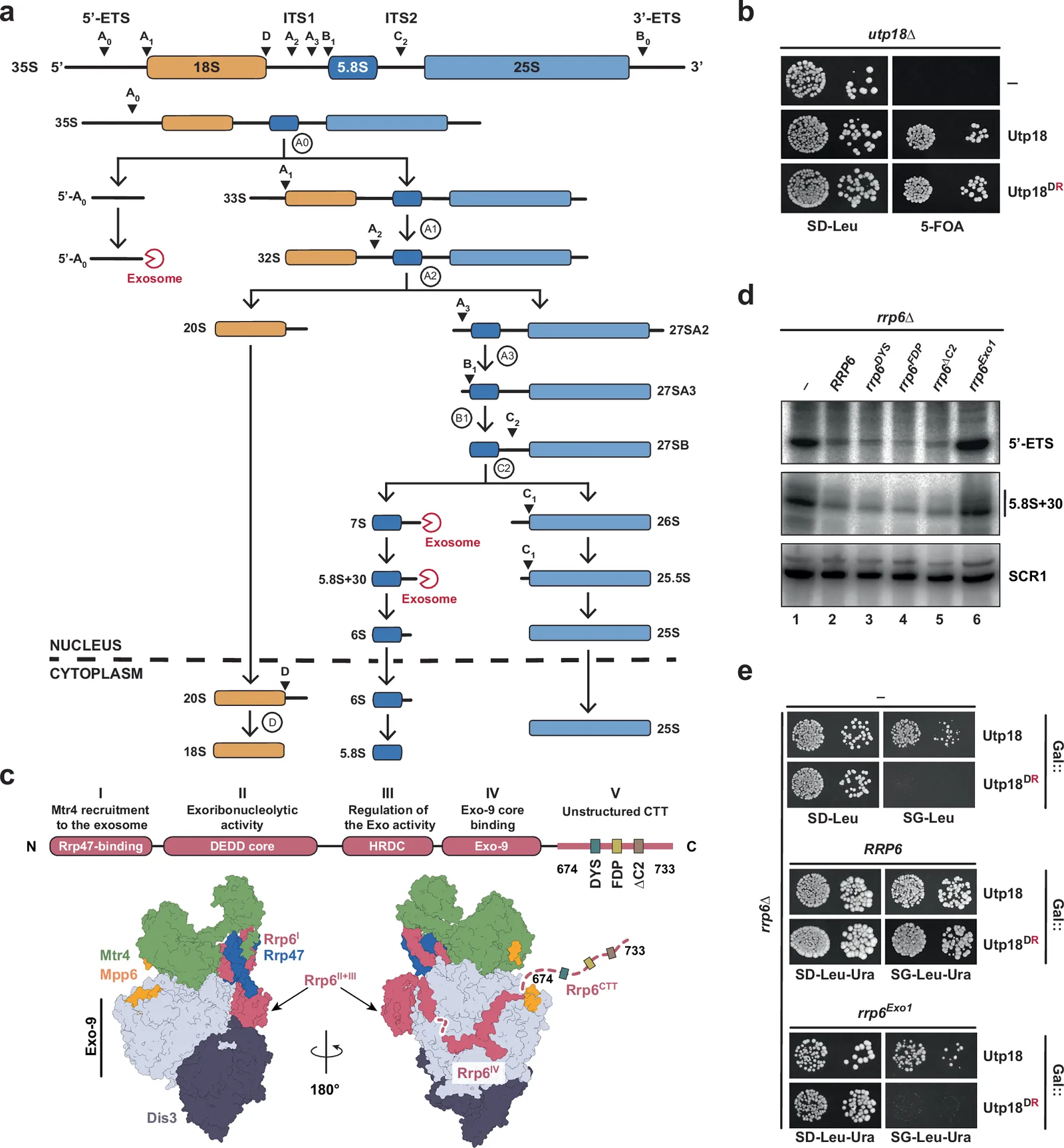
Rrp6 activity is required for yeast 5’-ETS RNA degradation. **a** Overview of pre-rRNA processing pathway in yeast. The spacer (5’-A0 ETS RNA) and pre-rRNAs (7S and 5.8S + 30) respectively degraded and processed by the RNA exosome, are indicated. **b** Growth analysis of yeast strains expressing wild-type Utp18 or the AIM mutant (Utp18^DR^). Indicated constructs were transformed into a *utp18∆* strain. Transformants were spotted in 10-fold serial dilutions on SD-Leu and 5-FOA plates. **c** Upper panel: domain representation of Rrp6 highlighting its C-terminal tail (residues 674–733, CTT) with three regions contacting the 90S pre-ribosome (DYS, FDP, ∆C2). Lower panel: cryo-EM structure of the yeast RNA exosome (PDB: 6FSZ) in complex with cofactors. The catalytically inactive Exo-9 core is shown in light purple. The Dis3 nuclease (dark purple) is placed at the bottom, while Rrp6 (red), Rrp47 (blue), Mpp6 (orange), and Mtr4 (green) are positioned on top of Exo-9. **d** Northern blotting analysis of total RNA isolated from *rrp6*∆ cells complemented with wild-type Rrp6 or its different mutants. Cells were grown in SR/Suc-Leu medium at 30 °C and harvested after 3 h. The 5’-ETS and 5.8S + 30 pre-rRNAs were detected using appropriate probes. SCR1 RNA levels were used as a loading control. **e** Growth analysis of conditional expression of Utp18 and Utp18^DR^ in *rrp6*∆ strain containing empty vector (−), Rrp6 and the catalytic dead Rrp6^Exo1^ mutant. Utp18 and Utp18^DR^ expression was under the control of *GAL1* promoter (Gal::). Cells were spotted in 10-fold serial dilutions and grown at 30 °C on SD-Leu-Ura or SG-Leu-Ura plates. Source data are provided as a Source Data file.

In yeast, degradation of the 5’-ETS 5′-A0 segment requires the RNA exosome (Fig. 4a), which is recruited to the 90S pre-ribosome through interactions between Mtr4 and Utp18^AIM^ ^6,21,23,45^. Substituting the conserved aspartate residue (D88) in Utp18^AIM^ with arginine (Utp18^DR^) disrupted Mtr4 binding in vitro and impaired 5′-ETS RNA degradation in vivo^23^. However, the Utp18^DR^-expressing yeast mutant cells did not exhibit a significant growth defect (Fig. 4b)^23^, suggesting the existence of a redundant pathway to degrade 5′-ETS RNA. Cryo-EM reconstructions of the yeast 90S pre-ribosome^39^ placed Fcf2, the ortholog of DNTTIP2, near 5′-ETS RNA (Fig. 2a, left panel, inset), with its ~90-residue N-terminal extension (Fcf2^N^) positioned adjacent to the 3′ end. Despite this location, Fcf2^N^ lacks a discernible AIM or its variants (Supplementary Fig. 5a), and Y2H assays failed to detect an interaction between Fcf2 and the yeast and human Mtr4/MTR4-Arch (Fig. 2c, rows 1 and 4). These data do not support a redundant role for Fcf2^N^ to recruit the RNA exosome to the yeast 90S pre-ribosome.

**Fig. 5:**
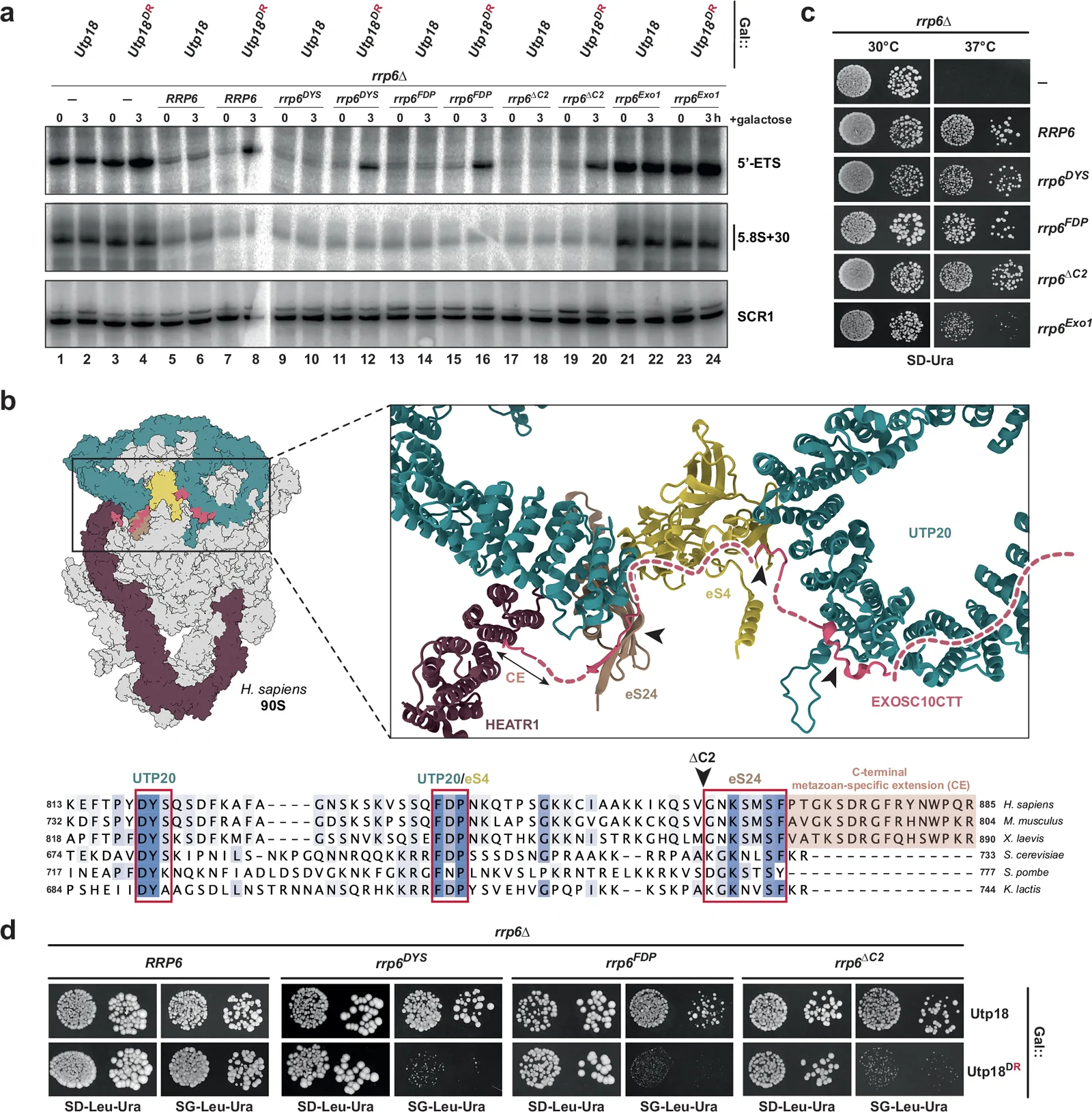
A role for Rrp6^CTT^ in yeast 5’-ETS RNA degradation. **a** Northern blotting analysis of total RNA isolated from *rrp6*∆ cells co-transformed with constructs expressing different Rrp6 and *GAL1*-driven Utp18 variants as indicated. Cells were grown in SR/Suc-Leu-Ura medium, induced with 2% galactose at OD_600_ 0.3, and harvested after 3 h of incubation at 30 °C. The 5’-ETS and 5.8S + 30 rRNAs were detected using appropriate probes. SCR1 RNA levels were used as a loading control. **b** Upper panel: human 90S pre-ribosome in the post-A1 stage (PDB: 7MQA) showing ribosomal proteins eS4 (yellow) and eS24 (light brown), assembly factors UTP20 (cyan) and HEATR1 (purple), and the C-terminal tail (CTT) of EXOSC10 (red). Black arrows indicate EXOSC10 regions interacting with the 90S pre-ribosome. Lower panel: sequence alignment of yeast Rrp6 and human EXOSC10, as well as other homologs from the indicated eukaryotic organisms. Conserved residues mediating interactions with the 90S pre-ribosome are framed in red. **c** Growth analysis of the *rrp6*∆ strain complemented with wild-type Rrp6 or different mutants. The strains were spotted in 10-fold serial dilutions and grown on SD-Ura at 30 °C and 37 °C. **d** Growth analysis of conditional expression of Utp18 and Utp18^DR^ in *rrp6*∆ strain complemented with wild-type Rrp6 or Rrp6^CTT^ mutants: Rrp6^DYS^, Rrp6^FDP^, and Rrp6^∆C2^. Utp18 and Utp18^DR^ expression was under the control of *GAL1* promoter (Gal::). Cells were spotted in 10-fold serial dilutions and grown at 30 °C on SD-Leu-Ura or SG-Leu-Ura plates. Source data are provided as a Source Data file.

Rrp6, the yeast ortholog of EXOSC10, is a distributive 3′-5′ exonuclease associated with the nuclear RNA exosome^22^. It comprises five functional domains: (I) an N-terminal domain that binds its stabilizer Rrp47 and Mtr4, linking the helicase to the exosome; (II) a globular catalytic DEDD core; (III) a regulatory HRDC domain; (IV) a C-terminal domain that interacts with the Exo-9 barrel; and (V) a C-terminal tail (CTT) that is conserved in unicellular and multicellular eukaryotes (Fig. 4c). Consistent with previous reports^6,46^, Northern blotting of total RNA extracted from *rrp6*∆ and catalytically inactive Rrp6^Exo1^ cells revealed elevated levels of 5′-ETS RNA (Fig. 4d, lanes 1 and 6). Notably, conditional expression of Utp18^DR^, but not wild-type Utp18, in these mutant strains was dominant lethal (Fig. 4e) and exacerbated 5′-ETS RNA accumulation (Fig. 5a, lanes 2, 4 and 22, 24). These data argue for a synergistic role for Rrp6 activity and the Mtr4-Arch:Utp18^AIM^ contact to efficiently degrade 5′-ETS RNA.

Cryo-EM structures of the human 90S pre-ribosome in different conformational states implicated EXOSC10 activity in degrading part of the 5’-ETS RNA^38,47^. These studies implicated a major structural reorganisation of the 90S pre-ribosome following cleavage at the A1 site, which permits EXOSC10^CTT^ to engage in multivalent contacts with ribosomal proteins eS4 and eS24 and assembly factors UTP20 and HEATR1 (Fig. 5b, upper panel, inset). Notably, the residues of EXOSC10^CTT^ involved in these contacts are conserved in all eukaryotes, apart from the HEATR1-contacting sequence that is found only in multicellular eukaryotes (Fig. 5b, lower panel). We investigated the functional contribution of these contacts to 5’-ETS RNA degradation by generating different Rrp6^CTT^ mutants in yeast: (1) the Rrp6^DYS^ mutant wherein the DYS residues that contact Utp20 were substituted to Ala; (2) the Rrp6^FDP^ mutant wherein the FDP residues that contact the Utp20:eS24 interface region were substituted to Ala; and (3) the Rrp6^∆C2^ mutant wherein the terminal -GNKSMSF- residues that contact eS24 were removed. In contrast to the rrp6∆ and catalytic dead Rrp6^Exo1^ mutant strains, which are temperature sensitive (Fig. 5c), mutant strains expressing the Rrp6^DYS^, Rrp6^FDP^ and the Rrp6^∆C2^ alleles grew at nearly wild-type rates (Fig. 5c). However, conditional expression of Utp18^DR^, but not Utp18, induced a strong dominant negative growth defect in the Rrp6^DYS^-, Rrp6^FDP^- and the Rrp6^∆C2^-expressing mutant strains (Fig. 5d). Northern blotting analyses of total RNAs extracted from the different mutant strains showed that unlike the *rrp6∆* and Rrp6^Exo1^-expressing mutants, the Rrp6^DYS^-, Rrp6^FDP^- and the Rrp6^∆C2^-expressing mutants alone did not accumulate 5’-ETS RNA and 5.8S + 30 pre-RNAs (Fig. 4d). Consistent with growth assays (Fig. 5d), Northern blotting analyses indicated a strong accumulation of 5’-ETS RNA, but not 5.8S + 30 pre-RNA, upon conditional expression of Utp18^DR^ in the Rrp6^DYS^ (Fig. 5a, lanes 10 and 12), Rrp6^FDP^ (Fig. 5a, lanes 14 and 16) and the Rrp6^∆C2^ (Fig. 5a, lanes 18 and 20) mutants. Based on all these studies, we suggest that establishing multiple contacts between the RNA exosome (Utp18^AIM^ and Rrp6^CTT^) and the 90S pre-ribosome is critical to initiate 5’-ETS RNA degradation.

### EXOSC10 and DNTTIP2^AIM^ cooperate to degrade human 5′-ETS RNA

**Fig. 6:**
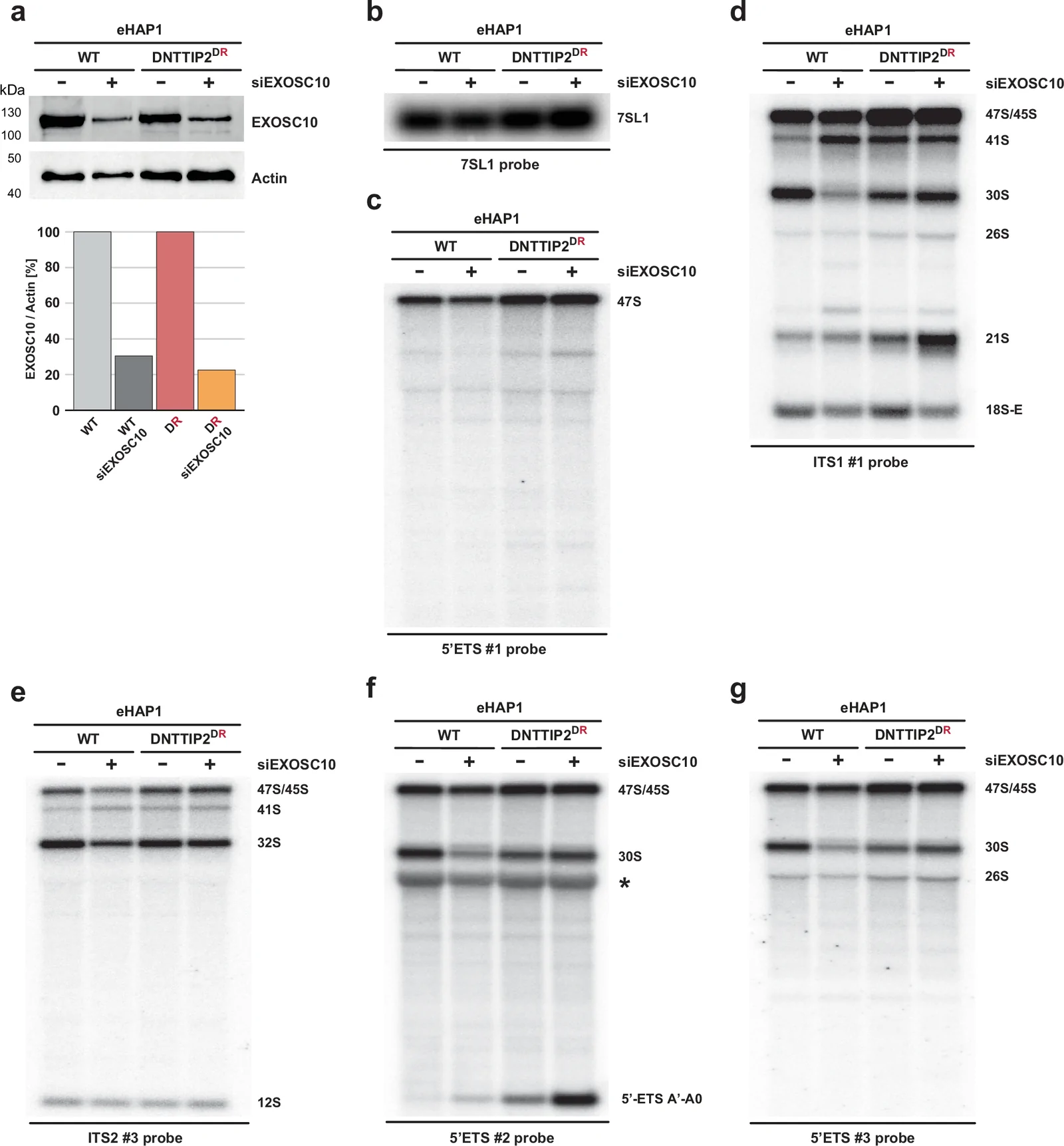
DNTTIP2^AIM^ and EXOSC10 cooperate to degrade 5’-ETS A’-A0 RNA. A control eHAP1 cell line expressing wild-type DNTTIP2 (C1) and a mutant cell line expressing the DNTTIP2^DR^ variant (M1) were transfected with siRNAs targeting EXOSC10 mRNAs, grown for 48 h and harvested. **a** Whole-cell lysates derived from the indicated cell lines were analysed by Western blotting (upper panel) using antibodies directed against EXOSC10 or actin (loading control). Lower panel depicts quantification of the Western blot signals (**b**–**g**) Total RNA extracted from these cells was analysed by Northern blotting using the following probes: **b** 7SL1 (loading control), **c** 5’ETS #1, **d** ITS1 #1, **e** ITS2 #3, **f** 5’ETS #2, and **g** 5’ETS #3. In each case, the rRNA precursors detected by the probes are indicated on the right. The asterisk marks a cross-hybridizing band corresponding to the mature 28S rRNA. The precise positions of the probes on the pre-rRNA are shown in Fig. 3a. Source data are provided as a Source Data file.

To investigate the functional interplay of DNTTIP2^AIM^ and EXOSC10 activity during human 40S subunit assembly, we used RNA interference to deplete EXOSC10 in control wild-type cells (clone C1) and DNTTIP2^DR^-expressing cells (clone M1). Western blotting analysis confirmed that EXOSC10 levels were reduced to ~30% of mock-transfected controls at 48 h post-siRNA transfection in wild-type and DNTTIP2^DR^-expressing lines (Fig. 6a). Total RNA was extracted from the cell lines, separated by denaturing agarose gel electrophoresis, and subjected to Northern blotting using different probes (Fig. 3a) to detect (Fig. 6c–g) and quantify (Supplementary Fig. 7) different pre-rRNA intermediates. The levels of 7SL1 RNA were used as a loading control and normalization for the Northern blotting data (Fig. 6b). Northern blotting analysis using the 5’ETS #1 and ITS1 #1 probes showed that EXOSC10 depletion did not affect 47S/45S pre-rRNA levels in either cell type (Fig. 6c, d and Supplementary Fig. 7e). We found that 41S pre-rRNA accumulated in DNTTIP2^DR^-expressing cells and upon depleting EXOSC10 in wild-type cells (clone C1). However, EXOSC10 depletion in DNTTIP2^DR^-expressing cells did not exacerbate 41S pre-rRNA accumulation (Fig. 6d, e and Supplementary Fig. 7e, f). We next examined the levels of the A′-A0 fragment within 5′-ETS RNA using probe 5′-ETS #2. In wild-type control cells (clone C1), EXOSC10 depletion led to a close to 2-fold increase in the accumulation of the A′-A0 region of 5′-ETS RNA (Fig. 6f and Supplementary Fig. 7g) but not of the two other, upstream and downstream 5’-ETS RNA fragments (Fig. 6c, g). Strikingly, this accumulation was enhanced by 10-fold in DNTTIP2^DR^ cells upon EXOSC10 depletion as compared to control cells (Fig. 6f and Supplementary Fig. 7g), indicating a synergistic defect. These results demonstrate that efficient degradation of the A′-A0 RNA fragment requires EXOSC10 and an intact MTR4:DNTTIP2^AIM^ interaction.

### EXOSC10 and DNTTIP2^AIM^ cooperate to trim the 3’ end of 21S pre-rRNA

The 3’ end of 21S pre-rRNA within the human 40S pre-ribosome is trimmed to generate the 18S-E pre-rRNA by the RNA exosome/EXOSC10 through interactions with MTR4^42,48^. Yet, the adaptor protein responsible for targeting the RNA exosome to carry out this processing step is unknown. We investigated whether DNTTIP2^AIM^ and EXOSC10 function in this metazoan-specific processing step. To this end, we monitored 21S pre-rRNA levels, using probe ITS1 #1 by Northern blotting analyses of total RNA from wild-type control (clone C1) and DNTTIP2^DR^-expressing cells, with or without EXOSC10 depletion. In wild-type cells (clone C1), we observed a modest increase in 21S pre-rRNA and a concurrent reduction in 18S-E levels upon EXOSC10 depletion (Fig. 6d and Supplementary Fig. 7e). This phenotype was strongly exacerbated (increase of 70% as compared to control cells) when EXOSC10 was depleted in DNTTIP2^DR^-expressing cells (Fig. 6d and Supplementary Fig. 7e). Notably, the levels of the 12S pre-rRNA remained unchanged upon perturbation of the MTR4:DNTTIP2^AIM^ interaction or knockdown of EXOSC10 (Fig. 6e and Supplementary Fig. 7f), consistent with a previous report indicating that 12S pre-rRNA processing requires the 3’ to 5’ exonuclease ISG20L2^49^. These analyses show that the metazoan-specific MTR4:DNTTIP2^AIM^ interaction and EXOSC10 cooperate to trim the 3′-end of 21S pre-rRNA within 40S pre-ribosomes.

### Productive 3’ end trimming of 30S pre-rRNA by the EXOSC10-bound exosome

The 3′ end generated by cleavage at site 2 within ITS1 serves as the initial substrate for EXOSC10-mediated trimming (Fig. 3a). During 40S subunit assembly, this 3′ end can be produced through two alternative pathways (Fig. 3a): pathway I, in which cleavage of the 41S precursor generates the 21S pre-rRNA; and pathway II, in which cleavage of the early 45S precursor yields the 30S pre-rRNA. A 90S pre-ribosome containing the 30S pre-rRNA is expected to be less compact than the 40S pre-ribosome containing the 21S pre-rRNA, as the former represents earlier intermediates that retain the 5′-ETS scaffold RNA bound to multiple UTP complexes and assembly factors. Northern blotting analyses using ITS1 #1, 5′ETS #2, and 5′ETS #3 probes revealed a 2-fold decrease in 30S pre-rRNA levels in EXOSC10-depleted control cells (Fig. 6d, f, g and Supplementary Fig. 7e). Unexpectedly, DNTTIP2^DR^-expression suppressed this phenotype of EXOSC10 depletion. In cells expressing DNTTIP2^DR^, depletion of EXOSC10 was not associated with 30S pre-rRNA depletion (Fig. 6d, f, g and Supplementary Fig. 7e). We propose that the presence of EXOSC10 on the RNA exosome favours productive, distributive trimming of the 30S pre-rRNA. Following EXOSC10 depletion, DNTTIP2^AIM^ still loads the RNA exosome core, leading to pre-ribosome degradation, but this activity is blocked by DNTTIP2^DR^.

## Discussion

To extend the current view of how the human RNA exosome is recruited by adaptor proteins to selectively degrade RNA substrates, we performed an unbiased Y2H screen using the MTR4-Arch as bait. Beyond established interaction partners, we identified interactors of MTR4-Arch, including CLE7, LAS2, and CDC5L, potentially linking the RNA exosome to tRNA splicing-ligation and pre-mRNA splicing, and an unknown RNP implicated in lung tumorigenesis. These interactors lack discernible AIMs, suggesting that MTR4-Arch recognizes adaptor proteins with greater sequence and structural diversity than previously appreciated.

Focusing on DNTTIP2, we found that its canonical AIM (DNTTIP2^AIM^) plays a critical role during 40S subunit assembly. The DNTTIP2^AIM^ is embedded within an N-terminal extension that is present in all metazoans but absent from DNTTIP2 homologues in lower eukaryotes, including yeast. However, cryo-EM structures of the human 90S pre-ribosome placed the DNTTIP2^AIM^ in a position analogous to yeast Utp18^AIM^, which recruits the Mtr4:RNA exosome complex for 5′-ETS RNA degradation. Human UTP18 lacks an AIM, suggesting that DNTTIP2 has replaced UTP18 as the dedicated adaptor for this critical degradation step. Our functional studies reinforce this model. Mutation of a single conserved aspartate within the DNTTIP2^AIM^ abolished binding to MTR4 and induced selective failure to degrade the A′-A0 fragment within 5′-ETS RNA, mirroring phenotypes observed in human cell lines expressing catalytic dead Rrp44/DIS3^50^. Human 5′-ETS RNA (~3000 nucleotides) is substantially longer than that of yeast (~600 nucleotides). Despite this divergence, cryo-EM studies indicate that Utp18^AIM^ in yeast and DNTTIP2^AIM^ in humans are positioned near the 3′ end of their RNA substrate. We also found that human 21S pre-rRNA 3′-end trimming after cleavage at site 2 requires the DNTTIP2^AIM^-docked MTR4:RNA exosome complex. This may favour spatial coordination of A′-A0 RNA degradation with 21S pre-rRNA trimming and safeguard the fidelity of ribosome assembly. Yeast pre-rRNA does not undergo this trimming step, and we speculate that relocation of the AIM reflects this change in the pre-rRNA processing pathway.

**Fig. 7:**
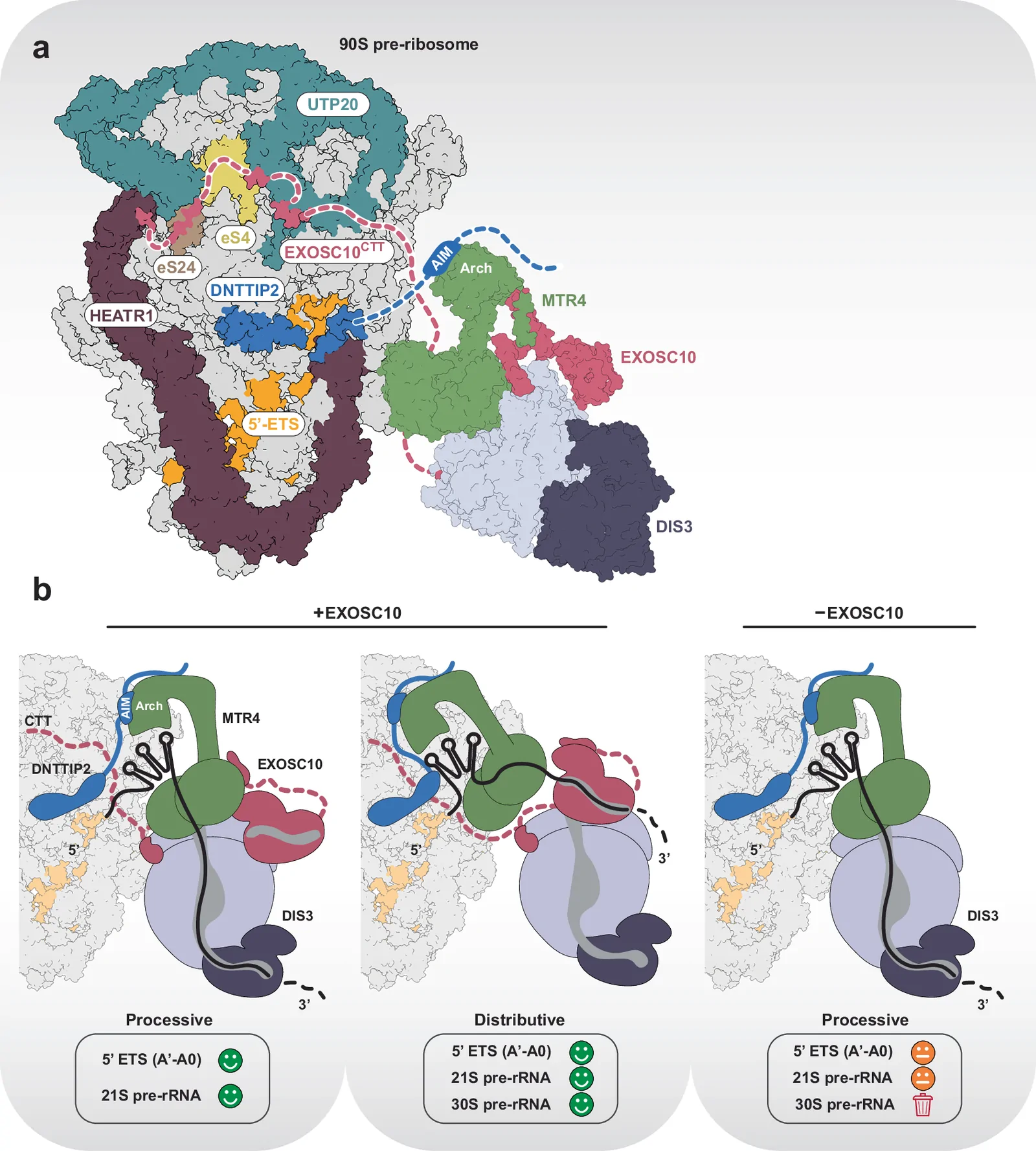
RNA processing within the 90S pre-ribosome mediated by the RNA exosome. **a** Schematic illustrating how the RNA exosome (PDB: 6FSZ) interacts with DNTTIP2^AIM^ via MTR4-Arch, and EXOSC10^CTT^ establishes multiple contacts with UTP20, HEATR1, and the r-proteins eS24 and eS4 within the 90S pre-ribosome (PDB: 7MQA and PDB: 7MQ8). **b** DIS3 and EXOSC10 function redundantly to process distinct RNA substrates. The 5′-ETS RNA and 21S pre-rRNA are processed in a processive manner (left) by channelling their 3′ ends through the exosome channel toward DIS3. EXOSC10 occludes the RNA exosome channel and mediates distributive processing of the 5′-ETS RNA, 21S pre-rRNA, and 30S pre-rRNA (middle). In the absence of EXOSC10, processing of the 5′-ETS RNA and 21S pre-rRNAs is impaired and becomes dependent on the processive activity of DIS3 (right). Productive trimming of the 30S pre-rRNA requires EXOSC10; without it, the molten 30S pre-rRNA is aberrantly channelled to DIS3 and degraded.

In yeast, redundant mechanisms recruit the RNA exosome to 90S pre-ribosomes. Utp18^AIM^ and the exonuclease Rrp6 act cooperatively to promote 5′-ETS RNA degradation. Impairment of Utp18^AIM^:Mtr4 interactions or *RRP6* deletion induced only modest 5′-ETS accumulation and growth defects, whereas combined inactivation of Utp18^AIM^ and *rrp6∆* showed synergistic defects. Structural studies provided a mechanistic rationale underlying this cooperation, as the EXOSC10^CTT^ engages with multiple r-proteins and assembly factors to tether the RNA exosome to the 90S pre-ribosome. The critical requirement for the conserved Rrp6^CTT^ contacts when Utp18^AIM^ is mutated suggests that all these interactions cooperate to optimally position the 3′-end of 5′-ETS RNA for degradation by the RNA exosome. Recently, AlphaFold-based modelling was employed to map interactions of Mtr4-bound RNA exosome with the yeast 90S pre-ribosome^51^. These in silico studies predicted contacts between disordered regions of Utp14 and the Mtr4-RecA2 domain as well as with the RNA exosome subunit Rrp40. Genetic analyses indicated synergistic interactions between Utp14 mutants and Utp18^DR^/Rrp6^CTT^ mutants, implicating Utp14 as an additional scaffold coordinating RNA exosome access to 5′-ETS RNA and/or for pre-ribosome surveillance. The principle of redundant contacts also extends to metazoans (Fig. 7a). DNTTIP2^AIM^ and EXOSC10 collaborate in 5’ETS A’-A0 fragment degradation and 3′-end trimming of 21S pre-rRNA. We propose a conserved strategy in which adaptor-mediated recruitment of the Mtr4/MTR4:RNA exosome complex is reinforced through Rrp6^CTT^/EXOSC10^CTT^, creating a multivalent network of contacts (Fig. 7a, b, left panel). These ensure that degradation and trimming are initiated only when the 90S pre-ribosome has attained a suitable conformation. Such a system may serve as a checkpoint, coupling RNA decay and processing to ongoing maturation events within the 90S pre-ribosome^52^.

Notably, EXOSC10 depletion in wild-type cells strongly reduced 30S pre-rRNA levels, but this was not the case in DNTTIP2^DR^-expressing cells. Based on these observations and published structural data, we postulate that the presence of EXOSC10 on the RNA exosome channel favours productive distributive 3’ trimming by the DNTTIP2^AIM^-docked exosome^1,22,52^. In the absence of EXOSC10, DNTTIP2^AIM^ recruits the exosome core, with 3′ end of nascent pre-rRNA now channelled toward Rrp44/DIS3 for processive degradation (Fig. 7b middle & right panels). Together, these findings reveal a critical requirement for regulating the processive exosome core and suggest that this feature can be harnessed for quality control^52,53^. When 30S pre-rRNA fails to undergo proper compaction, we speculate that the resulting particle is unable to correctly tether the EXOSC10 to the 90S pre-ribosome and seal the exosome channel. This may license the DNTTIP2^AIM^-docked exosome core to capture and feed the 3’ end of the defective precursor into the channel for processive degradation. Such an interplay between MTR4:AIM adaptors and EXOSC10^CTT^ may reflect a paradigm in which multivalent interactions with ribonucleoprotein complexes enable the RNA exosome to couple processing fidelity with surveillance.

## Methods

### Yeast strains and plasmids

The *Saccharomyces cerevisiae* strains used in this study are listed in Supplementary Table 1. Preparations of media, yeast transformations, and genetic manipulations were performed according to established procedures. Genomic disruptions were performed using standard yeast molecular biology^54^. All plasmids and oligonucleotides used in this study are listed in Supplementary Table 2 and 3, respectively. Plasmids containing human adaptor protein gene fragments were generated by GeneScript (Piscataway, USA) and codon-optimized for expression in the yeast host. All recombinant DNA work was performed using *Escherichia coli* TOP10 cells. Gene mutations were generated with the QuickChange II site-directed mutagenesis kit (Agilent Technologies). All cloned DNA fragments and mutagenized plasmids were verified by Sanger sequencing.

### Yeast two-hybrid screening (ULTImate Y2H) and studies

The Yeast Two-Hybrid screening was performed by Hybrigenics (Évry-Courcouronnes, France) using human MTR4-Arch (residues 579–842) as bait against a fragmented human placental cDNA library as prey. Positive interactions were identified and classified using standard yeast molecular biology tools^55,56^. Genes encoding desired proteins were cloned into either pLexA or pACT/pACT2.2 vectors, carrying a DNA Binding Domain (DBD) and GAL4 Activation Domain (AD), respectively. These vectors were transformed into the yeast NMY32 strain, which encodes a HIS3 reporter gene. Transformed NMY32 yeast cells were plated in synthetic dextrose medium (SD) lacking leucine and tryptophan (SD-Leu-Trp) and incubated at 30 °C for 3–4 days. The cells were spotted in a 10-fold serial dilution on SD-Leu-Trp and SD-His, followed by incubation at 30 °C for 3–4 days.

### Biochemical interaction studies

Plasmids encoding His_6_-tagged protein fragments (CLE7^206-244^, LAS2^487-533^, and proteinA-DNTTIP2^477-520^) were co-transformed and co-expressed with GST-tagged MTR4-Arch (residues 579–842) /KOW (residues 619–788) in *E. coli* BL21(DE3) cells. 1 litre cultures were grown at 37 °C until the OD_600_ reached 0.6–0.8, followed by induction with 0.5 mM IPTG and overnight incubation at 18 °C. After harvesting, cells were resuspended in lysis buffer containing 20 mM HEPES pH 7.5, 150 mM NaCl, 10 mM imidazole, 5 mM MgCl_2_, 0.1% Triton X-100, 1 mM PMSF, 1 mM DTT, and ½ tablet of cOmplete EDTA-free protease inhibitor cocktail (Roche). Cell lysis was performed using a microfluidizer (Microfluidics), and lysates were clarified by centrifugation at 40,000 × *g* for 30 min at 4 °C. The soluble fractions (7 ml per sample) were incubated with pre-washed 100 µl Ni-NTA Agarose beads (Qiagen) for 1 h at 4 °C with rotation. After gentle centrifugation, the unbound fractions were discarded, and the resin was washed 3 times with 5 ml wash buffer containing 20 mM HEPES, pH 7.5, 150 mM NaCl, 30 mM imidazole, 5 mM MgCl_2_, and 1 mM DTT. Bound proteins were eluted in 40 µl 2× LDS and heated up at 70 °C for 10 min. Proteins were separated on an SDS-PAGE gel, visualized by Coomassie Blue staining, or by Western blotting. To assess the stability of the MTR4-Arch:DNTTIP2^477-520^ complex, pull-down assays were also carried out using wash buffers containing increasing NaCl concentrations (150, 250, 500, and 700 mM). Uncropped and unprocessed SDS-PAGE gels / Western blots are provided in the Source Data file.

### RNA extraction and Northern blotting analyses

Human eHAP1 cells (Horizon Discovery) were used for total RNA extraction with TRI reagent. Following the addition of chloroform, samples were vortexed vigorously and centrifuged at 12,000 × *g* for 15 min at 4 °C. The aqueous phases were subjected to a second extraction using a 1:1 mixture of water-saturated phenol and chloroform. RNA was precipitated by adding an equal volume of isopropanol and incubating for 10 min at room temperature. Precipitated RNA was pelleted by centrifugation at 12,000 × *g* for 20 min at 2–8 °C, washed with 1 ml of 80% ethanol, air-dried, and resuspended in nuclease-free water. The same eHAP1 cells were used for the transient knockdown of EXOSC10 using a siRNA pool (Dharmacon). Transfections were performed by calcium phosphate precipitation. Northern blotting experiments from eHAP1 cell lines were conducted according to the previously described protocol^57^. In short, equal amounts of total RNA were combined with five volumes of Glyoxal loading buffer made of 6 ml DMSO, 2 ml deionized glyoxal, 1.2 ml 10X BPTE, 600 µl 80% glycerol, and 40 µl of 10 mg/ml ethidium bromide. Samples were heated for 1 h at 55 °C, followed by RNA separation on 1.2% agarose gels in 1X BPTE running buffer (100 mM PIPES, 300 mM Bis-TRIS, and 10 mM EDTA). After electrophoresis, gels were rinsed twice with water and soaked in 75 mM NaOH for 20 min at room temperature to partially hydrolyse RNA. Subsequently, gels were rinsed again with water and soaked twice in 0.5 M Tris-HCl, pH 7.4, with 1.5 M NaCl for 15 min each to neutralize the pH. As the final step, gels were soaked twice in 5X SSC for 10 min. Following this step, RNAs were transferred overnight to Amersham Hybond N+ membranes (Cytiva) using 5X SSC transfer buffer. Next, membranes were exposed to 0.125 J of 365 nm UV light to crosslink RNAs. After crosslinking, membranes were hybridized with ^32^P-labeled oligonucleotide probes in hybridization buffer (6X SSC, 5X Denhardt’s buffer, 0.5% SDS). Once hybridization was complete, radioactive membranes were placed on PhosphorImager screens, and the signals were captured using a Typhoon imager (GE Healthcare). Finally, RNA levels were quantified using Multi Gauge software v3.0 (FUJIFILM) for precise measurement and analysis. Yeast cells were pre-cultured in synthetic raffinose/sucrose medium lacking leucine and uracil at 30 °C until the OD_600_ reached 0.3. Next, galactose was added to the cultures at a final concentration of 2% to induce desired gene expression. Cell samples were then harvested after 3 h of induction, and total RNA was extracted^58^. After extraction, RNA samples were separated on 8% acrylamide/urea gels and transferred to BrightStar Plus membranes (Invitrogen) using standard blotting techniques^58^. The sequences of all DNA probes used for Northern blotting are listed in Supplementary Table 4. Uncropped and unprocessed Northern blot scans are provided in the Source Data file.

### Cell Proliferation analyses

Human cell proliferation was assessed using MTS assay (CellTiter 96 AQueous One Solution Cell Proliferation Assay, Promega). Data are presented as the mean of three technical replicates from at least three biological samples for each condition.

### Protein Synthesis SUnSET assay

Global protein synthesis was assessed using the SUnSET method^40^. Puromycin (InVivoGen) was added to the culture medium at a final concentration of 1 µM, and cells were incubated for 60 min at 37 °C. Following cell lysis, equal amounts of total proteins were analysed by Western blotting using anti-puromycin antibodies (Sigma Aldrich). Signal intensities were normalized according to the total protein content of each sample using Stain-Free gels (Bio-Rad).

### Protein structure modelling and sequence analysis

Single- and multi-chain protein structure predictions were generated using AlphaFold2 v1.5.5 via the Google CoLab platform^59,60^. All predictions were made using the MTR4-Arch domain and fragments of different adaptors identified in the Y2H screen. For each prediction, model confidence was assessed using the predicted Local-Distance Difference Test (pLDDT) scores, Predicted Aligned Errors (PAE), and pTM/ipTM scores^61–63^. The highest-confidence models were visualized using PyMOL v2.5.5^64^ or UCSF ChimeraX 1.8^65^ software. The generated AlphaFold2 models are provided as Supplementary Data 1–8. All protein sequences were retrieved from the SGD^66^ and Uniprot^67^ databases. Sequence alignments were generated using Jalview v2.11.4.1 and visualised using its integrated algorithm^68^.

### Statistics and reproducibility

Experiments in Figs. 1b, c, 2c, 3b–g, 4b, e, 5c, d, and 6a–g; Supplementary Fig. 1a, right panel; Supplementary Fig. 2a–d; Supplementary Fig. 6a–d were performed at least three times, and one representative result was used for figure preparation. Experiments in Figs. 4d and 5a were done twice, and one representative result was used for figure preparation. Quantification in Supplementary Figs. 6a, c and 7a–g are presented as mean (vertical columns) ± standard deviation (error bars). The individual data points are shown as black dots. Statistically significant differences were determined using Student’s *t* tests in Microsoft Excel (Microsoft Office, version 16).

### Reporting summary

Further information on research design is available in the Nature Portfolio Reporting Summary linked to this article.

## Supplementary information

Supplementary Information

Description of Additional Supplementary Files

Supplementary Data 1

Supplementary Data 2

Supplementary Data 3

Supplementary Data 4

Supplementary Data 5

Supplementary Data 6

Supplementary Data 7

Supplementary Data 8

Reporting Summary

Transparent Peer Review File

## Source data

Source Data

## Data Availability

All data generated or analysed in this study are included in the main text, figures, and Supplementary Information. Yeast strains, plasmids, and oligonucleotides used for cloning and Northern blotting are listed in Supplementary Tables 1–4. All plasmid maps are available upon request from the corresponding authors. AlphaFold2 predicted structures as PDB-format coordinate files are provided as Supplementary Data. Source data are provided with this paper.
