# Supplementary material for: DNTTIP2 coordinates RNA exosome activities to ensure fidelity of human ribosome assembly
Source: Nat Commun. 2026 Aug 7;17:9488. doi: 10.1038/s41467-026-76536-x (PMC13542088; doi:10.1038/s41467-026-76536-x)
Supplement: Supplementary file 2 [file 41467_2026_76536_MOESM12_ESM.pdf]

nature portfolio

Peer Review File

DNTTIP2 coordinates RNA exosome activities to ensure fidelity of human ribosome assembly

Corresponding Author: Professor Vikram Panse

This file contains all reviewer reports in order by version, followed by all author rebuttals in order by version.

Version 0:

Reviewer comments:

Reviewer #1

(Remarks to the Author)
Review NatComm

The RNA exosome is a critical molecular machine that processes or degrades cellular RNAs. One of the key question in the field is how the exosome is recruited to its RNA targets. This recruitment typically occurs through adaptor proteins that interact with the RNA helicase hMtr4 and its Arch domain. This article starts with a yeast 2-hybrid screen that extends the inventory of proteins interacting with human MTR4. Then they show that one of the proteins identified in the Y2H screem DNTTIP2 is the elusive adaptor that recruits the RNA exosome to the human 90S pre-ribosome to degrade 5′-ETS RNA. They show that efficient processing of the 47S pre-rRNA to 18S-E pre-rRNA requires intact hMTR4-Arch:DNTTIP2AIM interactions. This is an important study as it establishes an important step of the ribosomal RNA processing pathway and shows how the RNA exosome is recruited to one of its most important (quantitatively speaking) target. One of the strengths of the study is the use of mutant cell lines in which the conserved aspartate residue within DNTTIP2 Arch interacting motif is replaced with arginine, a mutation that disrupts interaction with basic residues on the hMTR4-Arch in vitro. The use of this system is very elegant as it allowed the authors to specifically address the role of the interaction between DNTTIP2 and hMTR4 independently from other functions.

I thought that the paper is very strong and presents a comprehensive story that is a great candidate for publication in Nature Communications. I would suggest addressing the following points prior to publication:

Most of the paper focuses on the role of the exosome in mediating rRNA processing in particular 5′-ETS RNA degradation. However the beginning of the paper presents the list of proteins identified through a 2-hybrid screen and discusses the interactions with hMTR4 of these different proteins, which have roles that are very different from rRNA processing (eg tRNA splicing). It seems to me that this part of the paper is tangential to the main story and that discussing these proteins not involved in rRNA processing distracts from the main message of the paper. I would suggest removing this part of the article to focus on the DNTTIP2 story. The authors can publish these other interactions in another paper, including the differences in binding modes. This would make the present article much more concise and focused.

The northern blots presented in most figures do not show any loading controls. This is problematic as the authors make conclusions re. accumulation of specific processing/degradation intermediates but it is difficult to evaluate these claims without loading controls. In blot that show multiple species (eg Fig.8e) it's not too much of an issue because some of the RNA species shown do not show any variations in levels, in contrast to others affected by cell line mutations. However in other figures (eg 6d, 7d, 8b), it is harder to evaluate the conclusions without proper loading controls. I understand that this is an issue when evaluating mutations that affect rRNA processing/levels, but an effort should be made to present proper loading controls for these experiments.

Some of the data presented for yeast (especially Figure 4,6,7) should be discussed and compared to a recent paper(Buzovetsky & Klinge, Nature 2025) . This article just came out while the current manuscript was being submitted and it is important that the authors compare the findings presented here to some of the experiments presented in that paper.

The paper is very lengthy – including the discussion. An effort should be made to make it more concise. Eliminating some of the data shown early would help (see above). However an effort should be made to rewrite the paper in a more concise way

and avoid repeating detailing most of the results in the discussion.

Minor Points.
Line 111-113. It is unclear how NRDE2 fits into the paragraph of Mtr4 Arch-interacting adaptors. Does NRDE2 belong to that category? If so the sentence should be modified to clearly state so.

Figure 1A. The authors show the crystal structure of hMTR4 highlighting the two Arginines important for canonical AIM recognition. This structure by itself is not very helpful, I would suggest instead showing the structure of the yeast Mtr4:Nop53AIM complex where these two arginines within the KOW subdomain form salt bridges with a conserved aspartate in the AIM, as mentioned later in the manuscript.

Paragraph line 157. Is it necessary to mention alpha fold prediction failures?

Figure 7D. The labeling of the figure should be modified to indicate Gal::Utp18 and mention that the 3hrs are in medium+galactose, otherwise it is difficult to understand this figure without having to refer to the legend.

Many of the figures show mutants of different factors (Utp18, Rrp6) but do not use the correct yeast nomenclature. For instance Rrp6DYS should be rrp6-DYS (italicized lower case for a mutant). The wild-type should be RRP6 (italicized, upper case).

Reference 27. contains “Accession Numbers GSE84172” – please correct.

Reviewer #2

(Remarks to the Author)
Summary
This manuscript investigates the recruitment of the human RNA exosome by MTR4 adaptor proteins, expanding the known repertoire to include a tRNA splicing ligase complex component (CLE7/RTRAF), a spliceosome-associated factor (CDC5L), and the nucleolar ribosome assembly factor DNTTIP2. The study identifies a canonical Arch-Interacting Motif (AIM) in DNTTIP2 that mediates binding to the hMTR4-Arch domain, facilitating selective degradation of the 5′-External Transcribed Spacer (5′-ETS) RNA during 40S ribosomal subunit maturation. Using CRISPR/Cas9-engineered eHAP1 cells carrying a DNTTIP2 AIM mutation, the authors demonstrate impaired 5′-ETS processing, delayed pre-rRNA cleavage, reduced 18S-E pre-rRNA levels, and decreased global protein synthesis. The manuscript further examines cooperation with EXOSC10 and provides structural insights into MTR4:AIM interactions, integrating data from both yeast and human systems.
Overall, the work addresses a central question in RNA biology: how the exosome achieves selective pre-rRNA processing in metazoans. While it presents compelling mechanistic insights, several key conclusions rely heavily on in vitro assays, computational modeling, or indirect evidence, and additional functional validation would strengthen the claims. Additionally, the manuscript would benefit from clearer organization to highlight the major findings. The text frequently shifts between yeast and human models, which can make the narrative difficult to follow. Although multiple new adaptors are identified, functional follow-through is largely restricted to DNTTIP2, leaving the roles of CLE7 and CDC5L less well defined. Greater clarity in linking each adaptor to its functional consequences would improve readability and emphasize the broader significance of the study.

Major Comments
1. Y2H Screen and hMTR4 Interactors

The authors used a yeast two-hybrid (Y2H) screen to identify hMTR4-Arch interacting proteins, reporting candidates including CLE7, LAS2, CDC5L, and DNTTIP2. However, evidence for these interactions is largely limited to Y2H and in vitro binding assays. AlphaFold2 failed to generate reliable structural models for CLE7, LAS2, and CDC5L. To strengthen the conclusions, the authors should:
- Validate these interactions using endogenous proteins in human cells, e.g., via co-immunoprecipitation.
- Test the requirement of AIM motifs by generating AIM-deleted mutants for each candidate.
- Demonstrate interaction with the RNA exosome in an MTR4-dependent manner using wild-type and mutant MTR4.

As currently presented, the functional relevance of CLE7, LAS2, and CDC5L remains speculative. Either additional functional validation should be included, or the limitations clearly discussed.

2. DNTTIP2 as the Metazoan 5′-ETS Adaptor
The identification of DNTTIP2 as a metazoan 5′-ETS adaptor via its AIM is potentially significant. However, additional experiments are needed to consolidate this claim.

The authors should demonstrate that DNTTIP2, and specifically its AIM, is required for MTR4 binding to 5′-ETS RNA using RNA immunoprecipitation or a comparable in vivo assay.
Rescue experiments to test whether re-expression of wild-type DNTTIP2 restores pre-rRNA processing in mutant lines should be conducted.

While Northern blot analyses (Figure 5) indicate inhibited 5′-ETS degradation in DNTTIP2^D492R cells, the accumulation of 47S, 41S, and 21S pre-rRNAs, along with reduced 18S-E and 30S levels and decreased proliferation, could reflect general

ribosome biogenesis stress rather than a specific processing defect. Please comment.

Parallel validation using RT-qPCR could provide more quantitative support.

Additional points:
The subcellular localization of endogenous DNTTIP2 relative to the 90S pre-ribosome should be clarified.

The proposed role of EXOSC10 in counteracting uncontrolled processive degradation by the DNTTIP2-AIM-loaded exosome is based on indirect evidence. Further evidence to support these claims should be provided.

3. Manuscript Organization
The manuscript could be reorganized for clarity. The frequent switching between yeast and human systems makes the narrative difficult to follow. The authors should consider focusing primarily on DNTTIP2 and the human system, consistent with the title and main conclusions.

If comparative analysis with yeast AIMs is essential, it should be clearly framed as a comparative section; otherwise, yeast-specific data (e.g., UTP18 and Rrp6) could be moved or removed to improve readability.

Minor Comments
- M2 clone is not shown in cell proliferation assays and shows variability in pre-rRNA processing. Please clarify these results.

- In Supplementary Figure 4a, DNTTIP2^DR appears expressed at higher levels than WT. The legend notes that samples were not normalized for protein concentration. Given the growth defect of the mutant, its expression would be expected to be lower. Please clarify.

- There are issues with figure referencing:
In Figure 3c, lanes 13–16 are mentioned before lanes 7–12.
Figure 7d is cited before Figures 7a–c.
Supplementary Figure 5 is referred to only in the discussion, which is unconventional.

-In Figure 8a, the actin loading control shows variable expression. Please provide more quantitative data for EXOSC10 knockdown efficiency.

The manuscript presents an important advance in understanding human exosome recruitment to pre-ribosomal RNA. However, key claims, especially the role of DNTTIP2 as a 5′-ETS adaptor, require additional experimental validation (rescue experiments, endogenous interactions, RNA binding, localization) and clarification of mechanistic interpretations. With these improvements, the study could make a strong contribution to the field.

Reviewer #3

(Remarks to the Author)
In this manuscript, Pisano and co-workers investigate how the RNA exosome is specifically recruited to the human 90S pre-ribosome to ensure the proper degradation and processing of the 5′-ETS and ITS1-containing rRNA pre-cursors during 40S biogenesis. Through a yeast two-hybrid screen using the human MTR4 Arch domain as bait, the authors identify several new hMTR4 interactors, including DNTTIP2. Intriguingly, in contrast to its yeast orthologue Fcf2, DNTTIP2 contains a canonical Arch-interacting motif (AIM). Structural modeling, biochemical assays, and CRISPR-generated AIM-deficient DNTTIP2 mutants (DNTTIP2D492R) establish that the AIM-mediated DNTTIP2:MTR4 interaction is necessary for human 5′-ETS RNA and acts synergistically with EXOSC10. Further, the authors demonstrate that the DNTTIP2:MTR4 interaction and EXOSC10 cooperate to trim the 3´-end of the 21S rRNA. By contrast, EXOSC10 depletion did not further reduce 3´-end trimming of 30S in DNTTIP2D492R expressing cells. From these data they conclude that productive pre-ribosomal RNA trimming within the 90S pre-ribosome necessitates EXOSC10 to prevent uncontrolled processive degradation by the DNTTIP2AIM-docked exosome core.
Altogether, the manuscript reveals important novel insights into exosome recruitment and rRNA processing in human cells. The identification of DNTTIP2 as an hMTR4 adaptor for exosome recruitment is a significant finding. The elaborate comparison between the scenario in yeast (where the AIM in Utp18 recruits Mtr4) and human cells is strong and convincing. The experiments on AIM-dependent rRNA processing by DNTTIP2 and the cooperation with EXOSC10 are technically sound and conclusive. However, to my opinion, the proposed model, which distinguishes productive RNA maturation from surveillance-mediated decay does not become entirely clear. The data do strongly support a model of multivalent and coordinated exosome docking, but a summarizing figure would greatly help to better illustrate this key point. The data provided in Figure 1 and Figure 2 of the manuscript rather distract from the key finding of the manuscript, which is the identification of DNTTIP2 as a novel AIM-containing MTR4 adaptor. I also feel that the discussion part on the newly identified hMTR4-Arch adaptors is a bit lengthy, as the data are purely descriptive and again distract from the main points of the discussion.
Comments:

1) The identification of Figure 1 and 2 hMTR4-Arch adaptors by Y2H assays is interesting, but I do feel that it somehow distracts from the key findings. Therefore, I would suggest to move these data to the Supplementary material.

2) Along this line, I also suggest to shorten this part in the discussion section.

3) The Northern Blot data (Figure 5, 8) look convincing, but show only single experiments. The authors mention in the methods section that experiments were performed at least three times. Showing statistics with quantitative data from these replicates would make the results even stronger.

4) In Figure 8a the authors mention depletion of EXOSC10 to 30% without providing proper quantification.

5) Generally, the data nicely show that DNTTIP2D492R expressing cells exhibit a growth defect correlating with impaired rRNA processing. Providing direct evidence that these cells fail to properly generate mature 40S ribosomes would further strengthen the data.

6) As mentioned above, a summarizing scheme illustrating the main finding and the concept of coordinated exosome docking would make it easier to capture the proposed model.

Version 1:

Reviewer comments:

Reviewer #1

(Remarks to the Author)
In this revised manuscript, the authors have addressed many of my concerns, and also those of the other reviewers of the original manuscript.The authors have responded to the points raised satisfactorily and have provided a revised version that properly addressed the issues that were raised. In particular, one of the major issue in the original manuscript was the lack of loading controls on the northern blots shown in several figures, and the lack of quantitative analysis. The authors have responded very conscientiously and provided revised figures which included proper loading controls and quantitative analysis. The authors have also addressed my other concerns appropriately and I would suggest accepting the manuscript for publication, assuming that the other reviewers also found the revised version to be satisfactory.

Reviewer #2

(Remarks to the Author)

Reviewer #3

(Remarks to the Author)
In their revised version, the authors adequately addressed my concerns. I therefore recommend publication of this manuscript.

Open Access This Peer Review File is licensed under a Creative Commons Attribution 4.0 International License, which permits use, sharing, adaptation, distribution and reproduction in any medium or format, as long as you give appropriate credit to the original author(s) and the source, provide a link to the Creative Commons license, and indicate if changes were made.
In cases where reviewers are anonymous, credit should be given to 'Anonymous Referee' and the source.
The images or other third party material in this Peer Review File are included in the article's Creative Commons license, unless indicated otherwise in a credit line to the material. If material is not included in the article's Creative Commons license and your intended use is not permitted by statutory regulation or exceeds the permitted use, you will need to obtain permission directly from the copyright holder.
To view a copy of this license, visit https://creativecommons.org/licenses/by/4.0/

Reviewer #1 (Remarks to the Author)

The RNA exosome is a critical molecular machine that processes or degrades cellular RNAs. One of the key question in the field is how the exosome is recruited to its RNA targets. This recruitment typically occurs through adaptor proteins that interact with the RNA helicase hMtr4 and its Arch domain. This article starts with a yeast 2-hybrid screen that extends the inventory of proteins interacting with human MTR4. Then they show that one of the proteins identified in the Y2H screem DNTTIP2 is the elusive adaptor that recruits the RNA exosome to the human 90S pre-ribosome to degrade 5′-ETS RNA. They show that efficient processing of the 47S pre-rRNA to 18S-E pre-rRNA requires intact hMTR4-Arch:DNTTIP2AIM interactions. This is an important study as it establishes an important step of the ribosomal RNA processing pathway and shows how the RNA exosome is recruited to one of its most important (quantitatively speaking) target. One of the strengths of the study is the use of mutant cell lines in which the conserved aspartate residue within DNTTIP2 Arch interacting motif is replaced with arginine, a mutation that disrupts interaction with basic residues on the hMTR4-Arch in vitro. The use of this system is very elegant as it allowed the authors to specifically address the role of the interaction between DNTTIP2 and hMTR4 independently from other functions. I thought that the paper is very strong and presents a comprehensive story that is a great candidate for publication in Nature Communications. I would suggest addressing the following points prior to publication:
Most of the paper focuses on the role of the exosome in mediating rRNA processing in particular 5′-ETS RNA degradation. However the beginning of the paper presents the list of proteins identified through a 2-hybrid screen and discusses the interactions with hMTR4 of these different proteins, which have roles that are very different from rRNA processing (eg tRNA splicing). It seems to me that this part of the paper is tangential to the main story and that discussing these proteins not involved in rRNA processing distracts from the main message of the paper. I would suggest removing this part of the article to focus on the DNTTIP2 story. The authors can publish these other interactions in another paper, including the differences in binding modes. This would make the present article much more concise and focused.
We appreciate the Reviewer's perspective and agree that DNTTIP2's role as the adaptor driving 5′-ETS RNA degradation is the primary focus of this manuscript. Our rationale for including the full set of adaptors identified in the screen was: (i) to demonstrate the diversity of factors linking MTR4 to distinct RNA processing pathways, and (ii) to provide a context for understanding how DNTTIP2 emerged as the long-sought adaptor dedicated to 5′-ETS RNA degradation. To make our work focused, we have moved the Y2H screen and its validation to Supplementary Information and have briefly discussed the binding properties of the additional identified adaptors. Comprehensive analyses of adaptors - particularly in the context of pre-tRNA and pre-mRNA splicing - is best addressed by specialized groups with expertise in those respective fields. Our goal in presenting these findings is to provide the community with validated starting points to accelerate progress in understanding the broader roles of MTR4 in RNA metabolism.

The northern blots presented in most figures do not show any loading controls. This is problematic as the authors make conclusions re. accumulation of specific processing/degradation intermediates but it is difficult to evaluate these claims without loading controls. In blot that show multiple species (eg Fig.8e) it's not too much of an issue because some of the RNA species shown do not show any variations in levels,

in contrast to others affected by cell line mutations. However in other figures (eg 6d, 7d, 8b), it is harder to evaluate the conclusions without proper loading controls. I understand that this is an issue when evaluating mutations that affect rRNA processing/levels, but an effort should be made to present proper loading controls for these experiments.
Thank you for this suggestion, we now provide loading controls for the Northern analyses using the abundant RNA component of signal recognition particle, the RNA Polymerase III-derived transcripts SCR1 in yeast (now Fig. 4d and Fig. 5a) and 7SL1 in human cells (now Fig. 3b and Fig. 6b) for pre-rRNA processing analyses. Levels of human pre-rRNAs were normalized to 7SL1 and we provide quantification and statistical analyses in Supplementary Figure 7.

Some of the data presented for yeast (especially Figure 4,6,7) should be discussed and compared to a recent paper(Buzovetsky & Klinge, Nature 2025) . This article just came out while the current manuscript was being submitted and it is important that the authors compare the findings presented here to some of the experiments presented in that paper.
Thank you for raising this point. Previously, Singh et al. Science 2021 (PMID: 34516797) employed cryo-EM to define how the EXOSC10 C-terminal tail (CTT) engages with the human 90S pre-ribosome, revealing contacts with assembly factors (UTP20 and HEATR1) and ribosomal proteins (eS24 and eS4). The functional relevance of these interactions, however, was not investigated. Given the conservation of EXOSC10-CTT/Rrp6-CTT and its binding surfaces, we employed yeast genetics to probe the in vivo importance of these contacts. We find that the individual Rrp6-CTT interaction sites become essential when combined with Utp18^{DR} allele, a mutant that is impaired in binding to yeast Mtr4 (Fig. 5d). Northern blotting studies demonstrate that the combined perturbation of any indivdual Rrp6-CTT contact and the Mtr4 contact impair 5′-ETS RNA degradation (Fig. 5a).
Buzovetsky & Klinge, Nature 2025 employed AlphaFold to map additional interactions of yeast Mtr4-bound RNA exosome with the 90S pre-ribosome. These *in silico* studies predicted contacts between Utp14 and Mtr4-RecA domain as well as the RNA exosome subunit Rrp40. Genetic analyses point to synergistic interactions between Utp14, Utp18 and Rrp6-CTT mutants, implicating Utp14 as an additional contact coordinating RNA exosome activity on the 90S pre-ribosome. However, the molecular basis underlying these interactions for e.g. impaired 5'-ETS RNA degradation and/or pre-ribosome surveillance remains unclear. By contrast, our study provides direct evidence (genetic and Northern blotting) that 5′-ETS RNA degradation requires redundant contacts between the 90S pre-ribosome and Rrp6-CTT/Mtr4-bound RNA exosome. We have cited and this work in the Discussion (pages 15/16).

The paper is very lengthy – including the discussion. An effort should be made to make it more concise. Eliminating some of the data shown early would help (see above). However an effort should be made to rewrite the paper in a more concise way and avoid repeating detailing most of the results in the discussion.
We appreciate the Reviewer's concern regarding length and redundancy. To address this, we have moved the Y2H screen and biochemical validation to Supplementary information (requested by the Editor and Reviewer 3). We have focused the Discussion on how DNTTIP2 functions in driving 5′-ETS RNA degradation, pre-RNA processing and surveillance. While the manuscript originates from a screen-based

approach, reporting the full set of adaptors is valuable, as they provide new candidates that link MTR4 with diverse RNA processing pathways.

Minor Points.

Line 111-113. It is unclear how NRDE2 fits into the paragraph of Mtr4 Arch-interacting adaptors. Does NRDE2 belong to that category? If so the sentence should be modified to clearly state so.

NRDE2 engages with MTR4 through two distinct interfaces. It contains a canonical AIM that binds to MTR4-Arch, classifying it as an Arch-interacting adaptor. Structural studies (Wang et al 2019 PMID: 30842217) have shown that NRDE2 possesses an additional segment that contacts RecA2, providing auxiliary stabilization, and presumably locking MTR4 in a closed conformation. We have revised the text (page 4) to clarify that NRDE2 is an AIM-containing MTR4 Arch-interacting adaptor with additional RecA2-mediated contacts.

Figure 1A. The authors show the crystal structure of hMTR4 highlighting the two Arginines important for canonical AIM recognition. This structure by itself is not very helpful, I would suggest instead showing the structure of the yeast Mtr4:Nop53AIM complex where these two arginines within the KOW subdomain form salt bridges with a conserved aspartate in the AIM, as mentioned later in the manuscript.

Since our study focuses on human MTR4, we have included the crystal structure of the MTR4:NVL^{AIM} complex (Supplementary Fig. 1a, left panel). This structure shows how the conserved arginines within the MTR4-Arch engage with the AIM by forming salt-bridge interactions with the critical aspartate residue. This provides a framework for AIM recognition, analogous to the yeast Mtr4:Nop53^{AIM} complex.

Paragraph line 157. Is it necessary to mention alpha fold prediction failures?

We believe that acknowledging the limitations of AlphaFold is important. We suspect that the AlphaFold based Mtr4-Arch:AIM complex modelling is biased toward only “known” canonical β-sheet:β-sheet interactions. Failure to predict experimentally validated Mtr4-Arch-containing complexes, indicate that alternative interaction modes and/or motifs exist but remain uncharacterized. Given the diverse substrate specificity of the RNA exosome and the small number of known adaptor proteins, identifying these gaps is important. Our findings highlight the need to expand the repertoire of motifs that mediate Mtr4-Arch recognition by structural studies.

Figure 7D. The labeling of the figure should be modified to indicate Gal::Utp18 and mention that the 3hrs are in medium+galactose, otherwise it is difficult to understand this figure without having to refer to the legend.

Gal::Utp18 or Gal::Utp18^{DR} and +Galactose are depicted (now Fig. 5a, 5d, also in Fig. 4e). We now state in the Legend that the cells were grown in SR/Suc -Leu-Ura medium, and then induced with Galactose (2%) and harvested after 3 hours of incubation at 30 °C for Northern analyses (page 27).

Many of the figures show mutants of different factors (Utp18, Rrp6) but do not use the correct yeast nomenclature. For instance Rrp6DYS should be rrp6-DYS (italicized lower case for a mutant). The wild-type should be RRP6 (italicized, upper case).

Thank you, we corrected these mistakes.

Reference 27. contains “Accession Numbers GSE84172” – please correct.

Thank you, we corrected this error.

Reviewer #2 (Remarks to the Author)

Summary
This manuscript investigates the recruitment of the human RNA exosome by MTR4 adaptor proteins, expanding the known repertoire to include a tRNA splicing ligase complex component (CLE7/RTRAF), a spliceosome-associated factor (CDC5L), and the nucleolar ribosome assembly factor DNTTIP2. The study identifies a canonical Arch-Interacting Motif (AIM) in DNTTIP2 that mediates binding to the hMTR4-Arch domain, facilitating selective degradation of the 5′-External Transcribed Spacer (5′-ETS) RNA during 40S ribosomal subunit maturation. Using CRISPR/Cas9-engineered eHAP1 cells carrying a DNTTIP2 AIM mutation, the authors demonstrate impaired 5′-ETS processing, delayed pre-rRNA cleavage, reduced 18S-E pre-rRNA levels, and decreased global protein synthesis. The manuscript further examines cooperation with EXOSC10 and provides structural insights into MTR4:AIM interactions, integrating data from both yeast and human systems.
Overall, the work addresses a central question in RNA biology: how the exosome achieves selective pre-rRNA processing in metazoans. While it presents compelling mechanistic insights, several key conclusions rely heavily on in vitro assays, computational modeling, or indirect evidence, and additional functional validation would strengthen the claims. Additionally, the manuscript would benefit from clearer organization to highlight the major findings. The text frequently shifts between yeast and human models, which can make the narrative difficult to follow. Although multiple new adaptors are identified, functional follow-through is largely restricted to DNTTIP2, leaving the roles of CLE7 and CDC5L less well defined. Greater clarity in linking each adaptor to its functional consequences would improve readability and emphasize the broader significance of the study.
While cellular RNA populations are diverse, the number of known adaptors that recruit the RNA exosome for targeted turnover and/or surveillance is surprisingly small. Our unbiased Y2H screen was designed to address this gap, and we identified new MTR4 interactors, which we validated through established reconstitution and mutational studies. These adaptors connect the RNA exosome to distinct cellular pathways, CLE7 to tRNA biogenesis and CDC5L to pre-mRNA splicing. Defining their non-canonical AIMs and the precise roles of each of the AIMs requires substantial structure-function analyses - efforts best pursued by experts in those respective fields. We focused on the mechanistic analysis of the canonical DNTTIP2^{AIM}, a choice that leverages the expertise of the Panse, Henras & Tollervey laboratories.

Major Comments
1. Y2H Screen and hMTR4 Interactors
The authors used a yeast two-hybrid (Y2H) screen to identify hMTR4-Arch interacting proteins, reporting candidates including CLE7, LAS2, CDC5L, and DNTTIP2. However, evidence for these interactions is largely limited to Y2H and in vitro binding assays. AlphaFold2 failed to generate reliable structural models for CLE7, LAS2, and CDC5L. To strengthen the conclusions, the authors should:
- Validate these interactions using endogenous proteins in human cells, e.g., via co-immunoprecipitation.
- Test the requirement of AIM motifs by generating AIM-deleted mutants for each candidate.
- Demonstrate interaction with the RNA exosome in an MTR4-dependent manner using wild-type and mutant MTR4.

As currently presented, the functional relevance of CLE7, LAS2, and CDC5L remains speculative. Either additional functional validation should be included, or the limitations clearly discussed.
We appreciate the Reviewer's suggestion to investigate MTR4 interactions with CDC5L, CLE7, and LAS2 in vivo to provide further support for Y2H analyses, biochemical reconstitution and mutational studies. Co-IPs cannot distinguish direct contacts from indirect association mediated by the Mtr4-bound RNA exosome. This was evident when we examined the MTR4 IP-MS datasets from Lubas et al. 2011, Mol. Cell (PMID: 21855801). These experiments co-enriched all established adaptors (NOP53, ZCCHC8, NVL, NRDE2, ZFC3H1) alongside our Y2H screen hits CDC5L and CLE7, together with numerous components of the ribosome assembly pathway, tRNA biogenesis, and pre-mRNA splicing machineries. We also note that in the Supplementary Table in Lubas et al., 2011 several proteins are annotated under their former gene names: NOP53 as GLTSCR2, CLE7 as C14orf166, and NRDE2 as C14orf102.

The MTR4-bound RNA exosome together with EXOSC10-CTT make multiple redundant contacts with the 90S pre-ribosome, rendering co-IP studies with the DNTTIP2^{DR} mutant difficult to interpret. Our reconstitution strategy - co-expression in *E. coli* - provides unambiguous evidence for direct binding. This is the case with DNTTIP2, CLE7, CDC5L, and LAS2 fragments. Critically, arginine substitutions in the MTR4-Arch selectively disrupt these interactions, demonstrating that DNTTIP2, CLE7, CDC5L, and LAS2 engage with MTR4-Arch. These arginine residues were defined by the Conti laboratory as diagnostic for AIM recognition by Mtr4-Arch (Lingaraju et al. 2019 Nat. Comm. PMID: 31358741). We therefore consider biochemical reconstitution a rigorous, orthogonal method for validating Mtr4-Arch interactions.

In contrast to the canonical DNTTIP2^{AIM} and established adaptors, AlphaFold failed to model how CLE7, CDC5L, and LAS2 fragments interact with MTR4-Arch. We suspect that AlphaFold is biased toward canonical β-sheet:β-sheet contacts that drive "known" Mtr4-Arch:AIM interactions. Failure of AlphaFold to predict experimentally validated Mtr4-Arch-containing complexes, indicate that alternative interaction modes and/or motifs exist but remain uncharacterized. Stuctural studies are therefore essential to determine how the uncharacterized, non-canonical AIMs present within CLE7, CDC5L, and LAS2 rely on the arginine resiudes within Mtr4-Arch for robust interactions. We have reported the validated interactors identified in the screen to accelerate mechanistic studies by the respective communities.

2. DNTTIP2 as the Metazoan 5′-ETS Adaptor
The identification of DNTTIP2 as a metazoan 5′-ETS adaptor via its AIM is potentially significant. However, additional experiments are needed to consolidate this claim. The authors should demonstrate that DNTTIP2, and specifically its AIM, is required for MTR4 binding to 5′-ETS RNA using RNA immunoprecipitation or a comparable in vivo assay.
Multiple lines of evidence support the conclusion that DNTTIP2 contains a canonical AIM responsible for human 5'-ETS RNA degradation.

(i) An unbiased Y2H screen (using MTR4-Arch as bait) identified a specific DNTTIP2 fragment that binds to MTR4-Arch, an interaction we validated biochemically by co-expression studies in *E. coli* followed by pulldown assays. We showed that this

interaction is robust, withstanding 700 mM NaCl. The Y2H screen has merit, since in addtion to DNTTIP2^{AIM}, it identified all AIM containing adaptors reported to date.

(ii) Y2H and biochemical studies identified a canonical AIM within DNTTIP2: mutation of the critical aspartate (DNTTIP2^{DR}) abolished MTR4 binding in vitro, as did the reciprocal substitutions of the conserved MTR4-Arch arginine residues defined by the Conti laboratory as diagnostic for AIM recognition (Lingaraju et al. 2019 Nat. Comm. PMID: 313587419). AlphaFold reliably predicted the DNTTIP2^{AIM}:Arch interaction, thus providing structural validation of the Y2H and biochemical data.

(iii) Finally, we investigated the functional relevance of DNTTIP2^{AIM}, using a CRISPR-generated DNTTP2^{DR} mutant cell line. Cryo-EM structures of the human 90S pre-ribosome resolved by the Klinge laboratory showed how DNTTIP2 is positioned next to the 3′ end of 5′-ETS RNA (Singh et al. 2021 PMID: 34516797). The AIM-containing loop is unresolved, which is also the case for yeast Utp18^{AIM} on the yeast 90S pre-ribosome (Lau et al., 2020 PMID: 33326748). Cells expressing DNTTIP2^{DR} accumulate the A′-A0 fragment of 5′-ETS RNA, phenocopying catalytic dead mutants of the RNA exosome-bound exonuclease DIS3/RRP44 (see Fig. 3b in Kobylecki et al., 2018 PMID: 30266864).
EXOSC10 depletion in cells carrying DNTTIP2^{DR} induced a strong synergistic accumulation of the A′-A0 fragment, indicating that the RNA exosome-bound distributive exonuclease EXOSC10 cooperates with the DNTTIP2^{AIM} to drive efficient 5′-ETS RNA turnover. This feature is conserved in yeast, where combining Utp18^{DR} with Rrp6 mutants causes synthetic lethality (growth tests) and synergistic accumulation of 5’-ETS RNA (Northern blotting) (this study; Thoms et al., 2015 PMID: 26317469). These data establish DNTTIP2 as the MTR4 adaptor required for human 5′-ETS RNA degradation.

Rescue experiments to test whether re-expression of wild-type DNTTIP2 restores pre-rRNA processing in mutant lines should be conducted.
To rigorously control for specificity, we implemented a dual CRISPR-based knock-in strategy rather than relying on re-expression systems, which introduce variability in protein expression levels and alter stoichiometry of complexes. Using wild-type and AIM-disrupting mutant donor templates, we generated two different sets of isogenic CRISPR-cell lines, in parallel. This approach allowed us to assess pre-rRNA processing under physiological expression conditions and avoids artifacts associated with overexpression-based rescue experiments. Endogenous knock-in is the gold standard for evaluating allele-specific effects because it preserves stoichiometry and regulatory contexts enabling definitive conclusions.

While Northern blot analyses (Figure 5) indicate inhibited 5′-ETS degradation in DNTTIP2^D492R cells, the accumulation of 47S, 41S, and 21S pre-rRNAs, along with reduced 18S-E and 30S levels and decreased proliferation, could reflect general ribosome biogenesis stress rather than a specific processing defect. Please comment.
5′-ETS RNA is not a passive by-product of rDNA transcription but a functional scaffolding RNA that recruits and organizes assembly factors on the nascent 47S/45S pre-rRNAs. Its timely degradation by the MTR4-bound RNA exosome machinery is essential for releasing these factors to allow productive formation of the 41S, 21S, and 30S pre-rRNA intermediates and to initiate subsequent rounds of 40S assembly. In DNTTIP2^{DR} cells, disruption of the MTR4:DNTTIP2^{AIM} interaction induces a specific

failure to degrade the A’-A0 fragment within 5′-ETS RNA, generating a bottleneck at the earliest step of 40S maturation. This blockade prevents the release of assembly factors, further impairing early 40S assembly steps. Importantly, the A′-A0 fragment accumulates in DNTTIP2^{DR} cells, and this defect is exacerbated upon EXOSC10 depletion, consistent with a specific role for the DNTTIP2^{AIM}:MTR4 interaction in 5′-ETS RNA turn over. A similar genetic and biochemical signature is observed in yeast, wherein 5′-ETS RNA accumulation in *utp18*^{DR} mutant is strongly enhanced in *rrp6Δ* or *rrp6-CTT* mutants (this study; Thoms et al., 2015, PMID: 26317469), underscoring the conservation and specificity of the mechanism that degrades 5’-ETS RNA. We note that the 60S processing pathway remains unaffected in DNTTIP2^{DR}-expressing cells. This makes it unlikely that the DNTTIP2^{DR} mutation induces a general ribosome biogenesis stress. The selective strong accumulation of A'-A0 fragment of 5’-ETS RNA is consistent with specific loss of DNTTIP2^{AIM}-mediated recruitment of MTR4 to the 90S pre-ribosome.

Parallel validation using RT-qPCR could provide more quantitative support.
We appreciate the Reviewer’s suggestion to incorporate RT-qPCR as an additional validation method. However, Northern blotting is the gold-standard approach for analysing pre-rRNA processing intermediates. This is because only Northern analysis simultaneously resolves and quantifies all intermediate species based on their defined sizes, allowing processing defects to be interpreted simultaneously within the full pathway. In contrast, RT-qPCR detects only predefined junctions and cannot distinguish between closely related intermediates (e.g., 47S vs. 45S, 41S vs. 30S, or 21S vs. 18S-E), nor can it reveal aberrant/unexpected processing products. Moreover, the extensive secondary structure of pre-rRNAs introduces variable reverse-transcription efficiencies that limit quantitative interpretation. For these reasons, Northern blotting is the appropriate method for the analyses presented in this study. To address the Reviewer’s concern, we now provide quantitative and statistical analyses for the key Northern blotting data in Supplementary Figure 7, which confirm our conclusions.

Additional points:
The subcellular localization of endogenous DNTTIP2 relative to the 90S pre-ribosome should be clarified.
The molecular environment and cellular localization of DNTTIP2 are well established. The cryo-EM structure of the human 90S pre-ribosome shows the location of DNTTIP2 on the 90S pre-ribosome next to the 3’-end of 5′-ETS RNA, in line with its role as an adaptor for the RNA exosome to degrade the A'-A0 fragment (Singh et al. Science 2021 PMID: 34516797).
The endogenous protein - also known as TDIF2 - localizes to the nucleolus, as reported in Fig.1 in Kotaro et al., Genes & Cells 2011 (PMID:21668587). The nucleolar localization of DNTTIP2 is reported in “The Human Protein Atlas”: (https://www.proteinatlas.org/ENSG00000067334-DNTTIP2/subcellular).
These observations support the conclusion that DNTTIP2 is appropriately localized to engage with the 90S pre-ribosome. We have specified the nucleolar localization of DNTTIP2 (page 6).

The proposed role of EXOSC10 in counteracting uncontrolled processive degradation by the DNTTIP2-AIM-loaded exosome is based on indirect evidence. Further evidence to support these claims should be provided.

Our model for how EXOSC10 counteracts uncontrolled processive degradation of the 30S pre-rRNA by the DNTTIP2^{AIM}-loaded RNA exosome is grounded on experimental findings and established structural studies.

(a) We observed that EXOSC10 depletion in wild-type cells resulted in strongly reduced levels of 30S pre-rRNA. However, EXOSC10 depletion in DNTTIP2^{DR}-expressing cells did not alter 30S pre-rRNA levels, which were restored to close to nearly wild-type levels. From these experiments, we infer that 30S pre-rRNA is vulnerable to degradation by the RNA exosome core recruited by DNTTIP2^{AIM} when EXOSC10 is absent. This supports the conclusion that the presence of EXOSC10 prevents the DNTTIP2^{AIM}-bound RNA exosome core from unleashing its processive activity on 30S pre-rRNA.

(b) Structural studies from the Conti laboratory (Schuller et al 2018 PMID: 29519915) have defined how the RNA exosome toggles between its distributive (Rrp6/EXOSC10) and processive (Rrp44/DIS3) activities. In particular, the presence of Rrp6/EXOSC10 prevents Mtr4/MTR4 from aligning with the central channel of the RNA exosome, thereby blocking processive 3′-5′ degradation by Rrp44/DIS3. This insight was summarised in a cartoon in a News & Views by Kummer & Ban (2021) (PMID: 30044080). The functional requirement for Rrp6/EXOSC10 in competing with Mtr4/MTR4 for covering the channel of the RNA exosome, however, was never explored. Our experiments support a model in which the AIM-docked RNA exosome becomes permissive for central-channel engagement, in the absence of Rrp6/EXOSC10, and therefore processively degrades 30S pre-rRNA.

Our model is supported by: (i) the specificity of the 30S pre-rRNA degradation phenotype when EXOSC10 is depleted, (ii) its dependence on the MTR4:DNTTIP2^{AIM} contact, and (iii) the established structural constraints governing processive and distributive activities of the RNA exosome established by work from the Conti laboratory. Hence, we had discussed our findings as a speculative model in the Discussion.

3. Manuscript Organization

The manuscript could be reorganized for clarity. The frequent switching between yeast and human systems makes the narrative difficult to follow. The authors should consider focusing primarily on DNTTIP2 and the human system, consistent with the title and main conclusions. If comparative analysis with yeast AIMs is essential, it should be clearly framed as a comparative section; otherwise, yeast-specific data (e.g., UTP18 and Rrp6) could be moved or removed to improve readability.

The Hurt group identified Utp18^{AIM} that recruits yeast Mtr4-bound RNA exosome for 5′-ETS degradation (Thoms et al. 2015 Cell PMID: 26317469). Because 5′-ETS RNA removal is essential for 40S biogenesis, the lack of a growth defect of the Utp18^{DR} mutant (Thoms et al. 2015 Cell PMID: 26317469) was unexpected and suggested the existence of a redundant pathway to recruit the RNA exosome. Using yeast genetics and Northern blotting, we demonstrate that Rrp6, via its conserved C-terminal tail (CTT), provides this mechanism to degrade 5'-ETS RNA by engaging multiple regions of the 90S pre-ribosome. These findings support a model in which Rrp6-CTT cooperates with Utp18^{AIM} to ensure 5′-ETS RNA degradation. This aspect is central to the interpretation of data that show cooperation between the metazoan specific DNTTIP2^{AIM} and EXOSC10 to degrade the human A'-A0 fragment within 5'-ETS RNA.

Minor Comments
- M2 clone is not shown in cell proliferation assays and shows variability in pre-rRNA processing. Please clarify these results.
We have added the cell proliferation data for the M2 clone (Supplementary Fig. 6b).

- In Supplementary Figure 4a, DNTTIP2^DR appears expressed at higher levels than WT. The legend notes that samples were not normalized for protein concentration. Given the growth defect of the mutant, its expression would be expected to be lower. Please clarify.
Actin protein levels were used as a loading control to normalize for DNTTIP2 protein levels (now Supplementary Fig. 6a). We present a quantification of these analyses. After normalization using actin protein levels, wild-type DNTTIP2 and $DNTTIP2^{DR}$ protein levels were found to be similar, and the apparent difference is not statistically significant.
This experiment was performed to investigate whether the $DNTTIP2^{DR}$ mutant is expressed at levels comparable to the wild-type and is not destabilized. Given these normalized protein levels, and the Northern blotting data, our findings show that the reduced growth rate of $DNTTIP2^{DR}$ expressing cells is not due to altered protein abundance, but instead reflects the mutant's impaired ability to recruit the MTR4:RNA exosome for 5′-ETS RNA degradation, which we directly demonstrate using in vitro binding studies (Fig. 1c).

- There are issues with figure referencing:
In Figure 3c, lanes 13–16 are mentioned before lanes 7–12.
Lane 7 in Fig. 3c (now Fig. 1c) demonstrates a direct interaction between hMTR4-Arch and $DNTTIP2^{477-520}$. This interaction withstands 700 mM NaCl salt treatment as shown in Lanes 13-16. We chose to demonstrate the interaction between the MTR4-Arch and $DNTTIP2^{477-520}$, then establish its robustness, and finally describe how the different mutational analyses disrupt this interaction (lanes 7–12).

Figure 7d is cited before Figures 7a–c.
We have reorganised the Figure (now Fig. 5) accordingly.

Supplementary Figure 5 is referred to only in the discussion, which is unconventional.
While the data presented in Supplementary Figure 5 is interesting, we agree with the Reviewer that its placement solely within the Discussion section is unconventional and detracts from the main message of the work. Accordingly, we have chosen to remove the Figure.

-In Figure 8a, the actin loading control shows variable expression. Please provide more quantitative data for EXOSC10 knockdown efficiency.
The point of this experiment (now Fig. 6a) was to evaluate EXOSC10 protein levels after treating wild-type DNTTIP2 and mutant $DNTTIP2^{DR}$-expressing cells with EXOSC10 siRNAs. We now present the quantification of the Western analyses for these two sets of experiments below (Fig. 6a).
In the first set, for wild-type DNTTIP2 expressing cells we compared EXOSC10 protein levels in lane 1 (- siRNA EXOSC10) and lane 2 (+ siRNA EXOSC10). Actin protein levels were used for normalization purposes. After normalization, we found that EXOSC10 protein levels show a 70 % decrease. In the second set, for $DNTTIP2^{DR}$-expressing cells we compared EXOSC10 protein levels in lane 3 (- siRNA EXOSC10)

and lane 4 (+ siRNA EXOSC10). Following normalization with actin protein levels, we found that EXOSC10 protein levels showed a 78 % decrease.

The manuscript presents an important advance in understanding human exosome recruitment to pre-ribosomal RNA. However, key claims, especially the role of DNTTIP2 as a 5′-ETS adaptor, require additional experimental validation (rescue experiments, endogenous interactions, RNA binding, localization) and clarification of mechanistic interpretations. With these improvements, the study could make a strong contribution to the field.
The key claim that DNTTIP2^{AIM} functions as an RNA exosome adaptor for the degradation of 5′-ETS RNA is supported by different biochemical and functional studies which are summarized on page 5/6, Point 2.

Reviewer #3 (Remarks to the Author):

In this manuscript, Pisano and co-workers investigate how the RNA exosome is specifically recruited to the human 90S pre-ribosome to ensure the proper degradation and processing of the 5′-ETS and ITS1-containing rRNA pre-cursors during 40S biogenesis. Through a yeast two-hybrid screen using the human MTR4 Arch domain as bait, the authors identify several new hMTR4 interactors, including DNTTIP2. Intriguingly, in contrast to its yeast orthologue Fcf2, DNTTIP2 contains a canonical Arch-interacting motif (AIM). Structural modeling, biochemical assays, and CRISPR-generated AIM-deficient DNTTIP2 mutants (DNTTIP2D492R) establish that the AIM-mediated DNTTIP2:MTR4 interaction is necessary for human 5′-ETS RNA and acts synergistically with EXOSC10. Further, the authors demonstrate that the DNTTIP2:MTR4 interaction and EXOSC10 cooperate to trim the 3´-end of the 21S rRNA. By contrast, EXOSC10 depletion did not further reduce 3´-end trimming of 30S in DNTTIP2D492R expressing cells. From these data they conclude that productive pre-ribosomal RNA trimming within the 90S pre-ribosome necessitates EXOSC10 to prevent uncontrolled processive degradation by the DNTTIP2AIM-docked exosome core.
Altogether, the manuscript reveals important novel insights into exosome recruitment and rRNA processing in human cells. The identification of DNTTIP2 as an hMTR4 adaptor for exosome recruitment is a significant finding. The elaborate comparison between the scenario in yeast (where the AIM in Utp18 recruits Mtr4) and human cells is strong and convincing. The experiments on AIM-dependent rRNA processing by DNTTIP2 and the cooperation with EXOSC10 are technically sound and conclusive. However, to my opinion, the proposed model, which distinguishes productive RNA maturation from surveillance-mediated decay does not become entirely clear. The data do strongly support a model of multivalent and coordinated exosome docking, but a summarizing figure would greatly help to better illustrate this key point. The data provided in Figure 1 and Figure 2 of the manuscript rather distract from the key finding of the manuscript, which is the identification of DNTTIP2 as a novel AIM-containing MTR4 adaptor. I also feel that the discussion part on the newly identified hMTR4-Arch adaptors is a bit lengthy, as the data are purely descriptive and again distract from the main points of the discussion.

Comments:

1) The identification of Figure 1 and 2 hMTR4-Arch adaptors by Y2H assays is interesting, but I do feel that it somehow distracts from the key findings. Therefore, I would suggest to move these data to the Supplementary material.
Thank you for this constructive suggestion, we have now moved Fig. 1 & 2 to Supplementary Information.

2) Along this line, I also suggest to shorten this part in the discussion section.
As requested, we have shortened this part of the Discussion section.

3) The Northern Blot data (Figure 5, 8) look convincing, but show only single experiments. The authors mention in the methods section that experiments were performed at least three times. Showing statistics with quantitative data from these replicates would make the results even stronger.

As requested, we have now quantified the key Northern blotting data of the study (Supplementary Figure 7). The analyses strengthen the RNA-exosome adaptor function of DNTTIP2^{AIM} and a synergistic role for EXOSC10 in 5'-ETS RNA turnover and 21S trimming.

4) In Figure 8a the authors mention depletion of EXOSC10 to 30% without providing proper quantification.
We now provide quantification for the EXOSC10 Western analyses which show a 30% decrease in protein levels upon siRNA-mediated depletion (Fig. 6a).

5) Generally, the data nicely show that DNTTIP2D492R expressing cells exhibit a growth defect correlating with impaired rRNA processing. Providing direct evidence that these cells fail to properly generate mature 40S ribosomes would further strengthen the data.
We performed sucrose density gradient sedimentation analyses using lysates derived from wild-type DNTTIP2 (clones C1 and C2) and DNTTIP2^{DR}-expressing (clones M1 and M4) cells (Supplementary Fig. 6d). Both independent mutant clones exhibit a marked decrease in the 40S:60S subunit peak ratio (peak heights or peak area) as compared to wild-type clones, indicating a reduction in mature 40S subunits. This shift in subunit balance provides direct evidence that DNTTIP2^{DR}-expressing cells are impaired in 40S ribosome assembly, consistent with the pre-rRNA processing defects and reduced cellular proliferation.

6) As mentioned above, a summarizing scheme illustrating the main finding and the concept of coordinated exosome docking would make it easier to capture the proposed model.
Thank you for this suggestion; we present a cartoon which depicts redundant contacts made by components of the RNA exosome (EXOSC10^{CTT} and MTR4) on the human 90S pre-ribosome (Fig. 7a). A cartoon depicting distributive RNA exosome activity (EXOSC10/Rrp6) in wild-type cells and processive activity of the RNA exosome core in EXOSC10-depleted cells during human 90S pre-ribosome maturation is also presented (Fig. 7b).
