# Supplementary material for: DNTTIP2 coordinates RNA exosome activities to ensure fidelity of human ribosome assembly
Source: Nat Commun. 2026 Aug 7;17:9488. doi: 10.1038/s41467-026-76536-x (PMC13542088; doi:10.1038/s41467-026-76536-x)
Supplement: Supplementary file 3 [file 41467_2026_76536_MOESM1_ESM.pdf]

Supplementary Information

DNTTIP2 coordinates RNA exosome activities to ensure fidelity of human ribosome assembly

Agnese Pisano, Jessie Bourdeaux, Jarosław Mazur, Florine Roses, Yves Romeo, Elisabeth Petfalski, Tobias von Arx, Daniela Portugal-Calisto, Michaela Oborská-Oplová, Cohue Peña, Clément Chapat, David Tollervey, Anthony K. Henras[#] & Vikram Govind Panse[#]

[#]Corresponding authors:
Anthony K. Henras (anthony.henras@utoulouse.fr)
Vikram Govind Panse (vpanse@imm.uzh.ch)

Table of contents:

1. Supplementary Tables 1 to 4 (p. 2 – 4)
2. Supplementary Figures 1 to 7 (p. 5 – 13)
3. Supplementary References (p. 13)

Supplementary Table 1. Yeast strains used in this study.

Strain	Genotype	Origin
NMY32	*MATa trp1 leu2 (lexAop)8-ADE2 LYS2::(lexAop)4-HIS3 URA3::(lexAop)8-lacZ GAL4*	Dualsystems Biotech AG
*utp*18Δ	*MATa ura3 his3 leu2 met15 TRP1 UTP18::KANMX + pRS316-UTP18*	This study
*rrp6*Δ	*MATa ura3 his3 leu2 met15 TRP1 RRP6::KANMX*	This study

Supplementary Table 2. Plasmids used in this study.

Plasmids	Relevant markers	Source
pACT2.2_NOP53$^{1-201}$	*GAL4 AD-NOP53 (1-201) 2μ LEU2 AMP*	This study
pACT2.2_ZCCHC8$^{150-191}$	*GAL4 AD-ZCCHC8 (150-191) 2μ LEU2 AMP*	This study
pACT2.2_NVL$^{1-370}$	*GAL4 AD-NVL (1-370) 2μ LEU2 AMP*	This study
pACT2.2_CLE7$^{206-244}$	*GAL4 AD-CLE7 (206-244) 2μ LEU2 AMP*	This study
pACT_LAS2$^{487-533}$	*GAL4 AD-LAS2 (487-533) 2μ LEU2 AMP*	This study
pACT2.2_CDC5L$^{579-743}$	*GAL4 AD-CDC5L (579-743) 2μ LEU2 AMP*	This study
pACT_ZFC3H1$^{1139-1321}$	*GAL4 AD-ZFC3H1 (1139-1321) 2μ LEU2 AMP*	This study
pACT2.2_DNTTIP2	*GAL4 AD-DNTTIP2 2μ LEU2 AMP*	This study
pACT2.2_DNTTIP2$^{477-520}$	*GAL4 AD-DNTTIP2 (477-520) 2μ LEU2 AMP*	This study
pACT2.2_DNTTIP2^{D492R}	*GAL4 AD-DNTTIP2 D492R 2μ LEU2 AMP*	This study
pACT2.2_DNTTIP2$^{477-520\ D492R}$	*GAL4 AD-DNTTIP2 (477-520) D492R 2μ LEU2 AMP*	This study
pACT2.2_Fcf2	*GAL4 AD-FCF2 2μ LEU2 AMP*	This study
pACT2.2_UTP18^{N}	*GAL4 AD*-human *UTP18 (1-117) 2μ LEU2 AMP*	This study
pACT2.2_Utp18^{N}	*GAL4 AD-UTP18 (1-152) 2μ LEU2 AMP*	This study
pACT_LargeT	*GAL4 AD-SV40 LARGET antigen 2μ LEU2 AMP*	Dual Systems
pLexA_MTR4	*LexA*-human *MTR4 2μ TRP1 AMP*	This study
pLexA_MTR4 Arch	*LexA*-human *MTR4 Arch (579-842) 2μ TRP1 KAN*	This study
pLexA_MTR4 KOW	*LexA*-human *MTR4 KOW (619-788) 2μ TRP1 AMP*	This study
pLexA_MTR4 ΔArch	*LexA*-human *MTR4 ΔArch* (Δ588-841) *2μ TRP1 AMP*	This study
pLexA_MTR4 Arch^{R658R}	*LexA*-human *MTR4 Arch (579-842) R658A 2μ TRP1 KAN*	This study
pLexA_MTR4 Arch^{R743E}	*LexA*-human *MTR4 Arch (579-842) R743E 2μ TRP1 KAN*	This study
pLexA_MTR4 ^{R658A}	*LexA*-human *MTR4 R658A 2μ TRP1 AMP*	This study
pLexA_MTR4 ^{R743E}	*LexA*-human *MTR4 R743E 2μ TRP1 AMP*	This study
pLexA_Mtr4 Arch	*LexA-MTR4 (609-874) 2μ TRP1 AMP*	This study
pLexA_LaminC	*LexA-LAMINC 2μ TRP1 AMP*	Dual Systems
pGEX-6P-1_MTR4 Arch	*GST*-human *MTR4 Arch (579-842) AMP*	This study
pGEX-6P-1_MTR4 KOW	*GST*-human *MTR4 KOW (619-788) AMP*	This study
pGEX-6P-1_MTR4 Arch^{R658A}	*GST*-human *MTR4 Arch (579-842) R658A AMP*	This study
pGEX-6P-1_MTR4 Arch^{R743E}	*GST*-human *MTR4 Arch (579-842) R743E AMP*	This study
pET47b_CLE7$^{206-244}$	*HIS_6-CLE7 (206-244) KAN*	This study
pET47b_LAS2$^{487-533}$	*HIS_6-LAS2 (487-533) KAN*	This study
pET47b_DNTTIP2$^{477-520}$	*HIS_6-proteinA-DNTTIP2 (477-520) KAN*	This study
pET47b_DNTTIP2$^{477-520\ D492R}$	*HIS_6-proteinA-DNTTIP2 (477-520) D492R KAN*	This study
pRS315_Utp18	*UTP18 CEN LEU2 AMP*	This study
pRS315_Utp18^{D88R}	*UTP18 D88R CEN LEU2 AMP*	This study
pRS316_Rrp6	*RRP6 CEN URA3 AMP*	This study
pRS316_Rrp6^{DYS}	*RRP6 D680A Y681A S682A CEN URA3 AMP*	This study

pRS316_Rrp6^{FDP}	*RRP6 F704A D705A P706A CEN URA3 AMP*	This study
pRS316_Rrp6$^{\Delta C2}$	*RRP6 (1-724) CEN URA3 AMP*	This study
pRS316_Rrp6^{Exo1}	*RRP6 D238A E240A CEN URA3 AMP*	This study
yEP351gal_Utp18	*P_{GAL1}-UTP18 LEU2 AMP*	This study
yEP351gal_Utp18^{D88R}	*P_{GAL1}-UTP18 D88R LEU2 AMP*	This study
pUC57_NOP53$^{1-201}$	*NOP53 (1-201) AMP*	GeneScript
pUC57_ZCCHC8$^{150-191}$	*ZCCHC8 (150-191) AMP*	GeneScript
pUC57_NVL$^{1-370}$	*NVL (1-370) AMP*	GeneScript
pUC57_CLE7$^{166-244}$	*CLE7 (166-244) AMP*	GeneScript
pUC57_LAS2$^{487-533}$	*LAS2 (487-533) AMP*	GeneScript
pUC57_CDC5L$^{410-743}$	*CDC5L (410-743) AMP*	GeneScript
pUC57_ZFC3H1$^{1139-1321}$	*ZFC3H1 (1139-1321) AMP*	GeneScript
pUC57_DNTTIP2	*DNTTIP2 AMP*	GeneScript
pUC57_MTR4	human *MTR4 AMP*	GeneScript
pUC57_UTP18	human *UTP18 AMP*	GeneScript

Supplementary Table 3. Oligonucleotides used in this study.

Name	Sequence 5'-3'
NOP53 1-201 NdeI F	AAAAAACATATGATGGCTGCTGGTGGTTCCG
NOP53 1-201 BamHI R	AAAAAAGGATCCTCATGGGTTGTCAGAAGCCCA
ZCCHC8 150-191 NdeI F	AAAAAACATATGGACCACAAGGTGGAAGAAAGC
ZCCHC8 150-191 BamHI R	AAAAAAGGATCCTCACGGGTTTTCATTCAGCAG
NVL 1-370 NdeI F	AAAAAACATATGATGAAGCCAAGACCAGCCG
NVL 1-370 BamHI R	AAAAAAGGATCCTCAGGTGATAGCATCGATTTCATCAAT
CLE7 206-244 NdeI F	AAAAAACATATGTTGTTGCACATTGAAGAACTAAGAG
CLE7 206-244 BamHI R	AAAAAAGGATCCTCATCTACCAACCTTACCTAATCTGTG
CLE7 206-244 BamHI F	AAAAAAGGATCCGTTGTTGCACATTGAAGAACTAAGAG
CLE7 206-244 NotI R	AAAAAAGCGGCCGCTCATCTACCAACCTTACCTAATCTGTG
LAS2 487-533 BamHI F	AAAAAAGGATCCAGTTTCTGAAGATGACTTCTCAAAG
LAS2 487-533 XhoI R	AAAAAACTCGAGTTACATCTTCGGAGAACGTTCAC
LAS2 487-533 SacI R	AAAAAAGAGCTCTTACATCTTCGGAGAACGTTCAC
CDC5L 579-743 NdeI F	AAAAAACATATGGAAATGATCACCATGTTGCACTAC
CDC5L 579-743 BamHI R	AAAAAAGGATCCTCATTCGATTTGGTCCCACAAGT
ZFC3H1 1139-1321 BamHI F	AAAAAAGGATCCACCTTGTCCATTTCGTCCGTATC
ZFC3H1 1139-1321 XhoI F	AAAAAACTCGAGTTAGTTTTCTGGCTGAAAAGCGTATTTG
DNTTIP2 BamHI F	AAAAAAGGATCCAATGGTTGTGACC
DNTTIP2 XhoI R	AAAAAACTCGAGTTAGTTACGAAATTTC
DNTTIP2 477-520 NdeI F	AAAAAACATATGGGTGACACCGGTAGCCTG
DNTTIP2 477-520 BamHI R	AAAAAAGGATCCTCATTTTTCTTCTTCGATAGCCACTTC
pA-DNTTIP2 477-520 BamHI F	AAAAAAGGATCCGATGAAAACCGCGGCTCTTGC
pA-DNTTIP2 477-520 NotI R	AAAAAAGCGGCCGCTTATTTTTCTTCTTCGATAGCCACTTC
DNTTIP2 D492R F	TTATTCGTACCACCCCGGGTATGAGCGC
DNTTIP2 D492R R	GTGGTACGAATAACAAACAGAGCATTGTCACAG
Fcf2 NdeI F	AAAAAACATATGATGGATCAAAGTGTGGAAGACT
Fcf2 BamHI R	AAAAAAGGATCCTCACCTCCGCTTCTTTCTCA
UTP18 1-117 NdeI F	AAAAAACATATGATGCCACCAGAGCGTAGAAG

UTP18 1-117 BamHI R	AAAAAAGGATCCTTATTCTTGAACACGCGGACCTC
Utp18 1-152 NdeI F	AAAAAACATATGATGACAATGGCAACAACCGC
Utp18 1-152 BamHI R	AAAAAAGGATCCTTACTCGTTATAGGAGGTTCTTAATTT
MTR4 SmaI F	AAAAAACCCGGGGATGGCTGATGCATTTGGTGAC
MTR4 SalI R	AAAAAAGTCGACTCACAGGTACAGGCTCGCTGCGAAAAC
MTR4 Arch EcoRI F	AAAAAAGAATTCGAAGAAATCAATCCGGAATATATGC
MTR4 Arch NotI R	AAAAAAGCGGCCGCCTAGGTACGCGCCTTTTTCAGTTC
MTR4 KOW EcoRI F	AAAAAAGAATTCAATGAAGAAAGCGTTGTGATCTAC
MTR4 KOW PstI R	AAAAAACTGCAGTCAGTCTTGAATACCCATGTCATCG
MTR4 KOW XhoI R	AAAAAACTCGAGTCAGTCTTGAATACCCATGTCATCG
MTR4 ∆Arch F	GAAAAGGCGCGTACCGTGCTGCAGAT
MTR4 ∆Arch R	CGCGCCTTTTCCAGCATATATTCCGGATTG
MTR4 R658A F	CGTTCCTGCAGCCAGGTGCTCTGGTGAAGGTTAA
MTR4 R658A R	TTAACCTTCACCAGAGCACCTGGCTGCAGGAACG
MTR4 R743E F	AGCGCGATCAGCAGCGTGGAGCTGTATATTCCGAAAGACC
MTR4 R743E R	GGTCTTTCGGAATATACAGCTCCACGCTGCTGATCGCGCT
Mtr4 Arch EcoRI F	AAAAAAGAATTCTTCTTCCAATTTCAAAACGTTATTT
Mtr4 Arch PstI R	AAAAAACTGCAGTCATGATTCTGAAATTTTGCGTTTCA
Utp18-500 bp NotI F	AAAAAAGCGGCCGCTAGGAGACTCCGATCCCTTTTT
Utp18+500 bp XhoI R	AAAAAACTCGAGTGAACCTAAAACCATTTGCCTGTC
Utp18-500 bp XbaI F	AAAAAATCTAGATAGGAGACTCCGATCCCTTTTTG
Utp18+500 bp PstI R	AAAAAACTGCAGTGAACCTAAAACCATTTGCCTGTC
Utp18 D88R F	GTTATTTTTTGTGCGCGATGGTGGTAACGAAGATAG
Utp18 D88R R	GTTACCACCATCGCGCACAAAAAATAACTGATCGTTGTT
Rrp6-500 bp NotI F	AAAAAAGCGGCCGCGCCTTTTTGCATCATAAGAGC
Rrp6+500 bp XhoI R	AAAAAACTCGAGGCCTAAACGGATACAAAAAATC
Rrp6 DYS-AAA F	GCTGTGGCTGCTGCAAAAATTCCGAATATCTTATCTAATAAG
Rrp6 DYS-AAA R	GGAATTTTTGCAGCAGCCACAGCATCTTTTTCCGTTACACC
Rrp6 FDP-AAA F	AAGGAGAGCCGCCGCATCATCTAGCGATAGTAATGGACC
Rrp6 FDP-AAA R	CTAGATGATGCGGCGGCTCTCCTTTTCTTTTGTTGTCTATTA
Rrp6 ∆C2 (K725Stop) F	GGAGGCCTGCCGCCTAAGGTAAGAATCTGT
Rrp6 ∆C2 (K725Stop) R	ACAGATTCTTACCTTAGGCGGCAGGCCTCC
Rrp6 Exo1 (D238A E240A) F	GCCGTTGCTCTTGCGCATCACGATTATAGGTC
Rrp6 Exo1 (D238A E240A) R	CGTGATGCGCAAGAGCAACGGCAATCTCTTTCG

Supplementary Table 4. Northern probes used in this study.

Name	Sequence 5'-3'
5'ETS #1	GGAGACGAGAACGCCTGACACG
5'ETS #2	AGGGTCCTCTGCGAGCGGGTCG
5'ETS #3	GGAAGAGCGGGCCGGGAGAAGA
ITS1 #1	CCTCGCCCTCCGGGCTCCGTTAATGATC
ITS2 #3	GCGCGACGGCGGACGACACCGCGGCGTC
7SL1	GCCTCCCGAGTAGCTGGGACTACAGG
yeast 5'ETS	CGCTGCTCACCAATGG
yeast 5.8S+30	GCGTTGTTCATCGATGC
SCR1	ATCCCGGCCGCCTCCATCAC

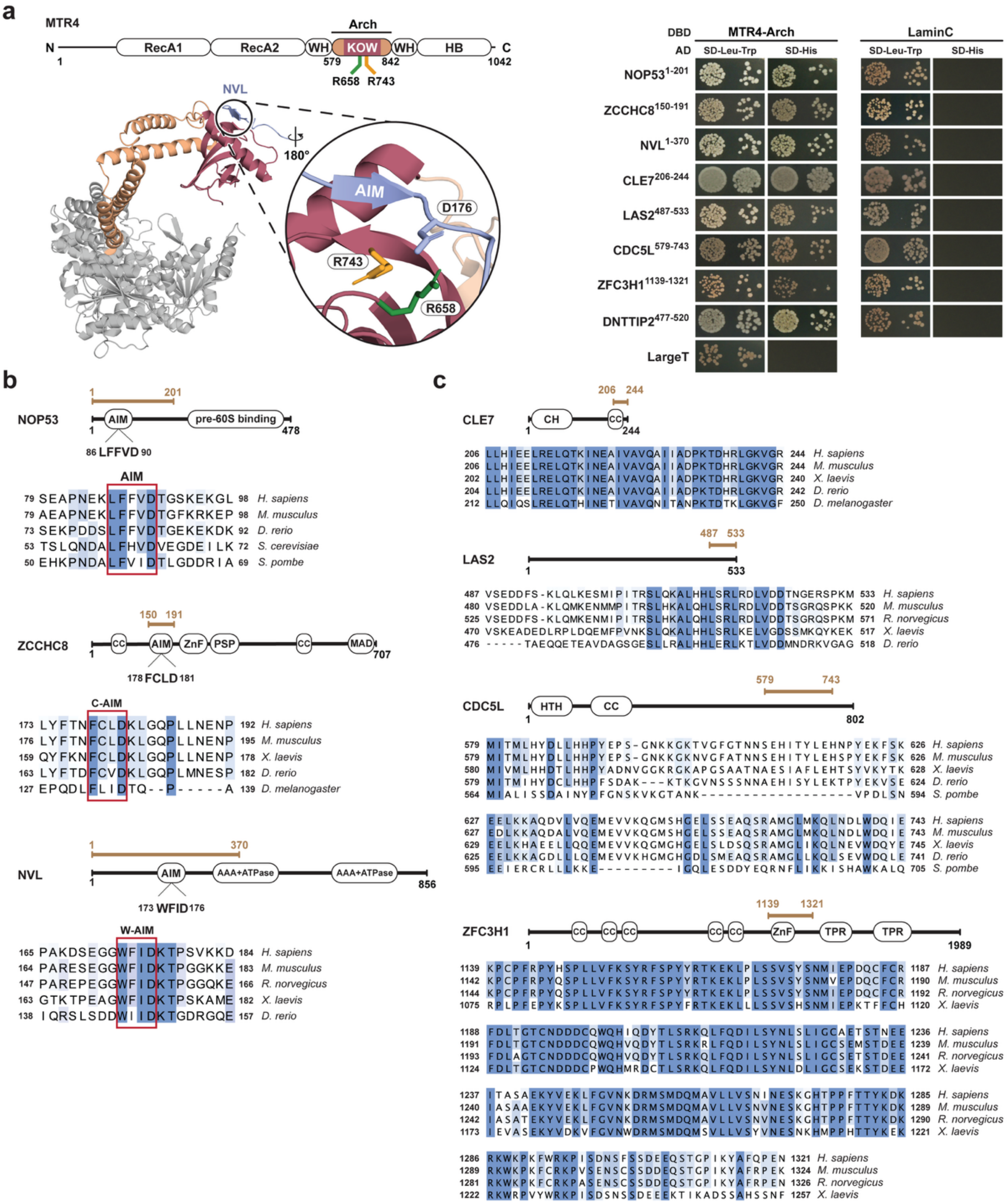

Supplementary Figure 1. Y2H screen identifies MTR4-Arch interactors. a Left panel: Domain organization and crystal structure of MTR4:NVL complex (PDB: 6RO1). The Arch and KOW domains of MTR4 are shown in orange and red, respectively. Two conserved arginine residues critical for canonical AIM recognition, R658 and R743, are marked in green and yellow, respectively. NVL and its AIM is shown in light purple. Right panel: Y2H analysis of MTR4-Arch interactions with identified proteins. Plasmids encoding the tested proteins fused to the LexA DNA-binding domain (DBD) and GAL4 activation domain (AD) as indicated were transformed into the NMY32 strain. Transformants were spotted on selective SD-Leu-Trp and SD-His media and incubated at 30 °C for 3-4 days. LaminC and LargeT were used as negative controls. (**b-c**) Domain representation and sequence alignments of identified MTR4 adaptor proteins containing **b** or lacking **c** canonical AIMs (framed in red). Fragments identified in the Y2H screen are marked in brown. AIM: Arch-Interacting Motif, CC: coil-coiled, ZnF:

zinc-binding domain, PSP: proline-rich domain, MAD: MTR4-activating domain, AAA^{+}: ATPase associated with diverse cellular activities, CH: calponin homology-like domain, HTH: Helix-Turn-Helix domain, TPR: tetratricopeptide repeat.

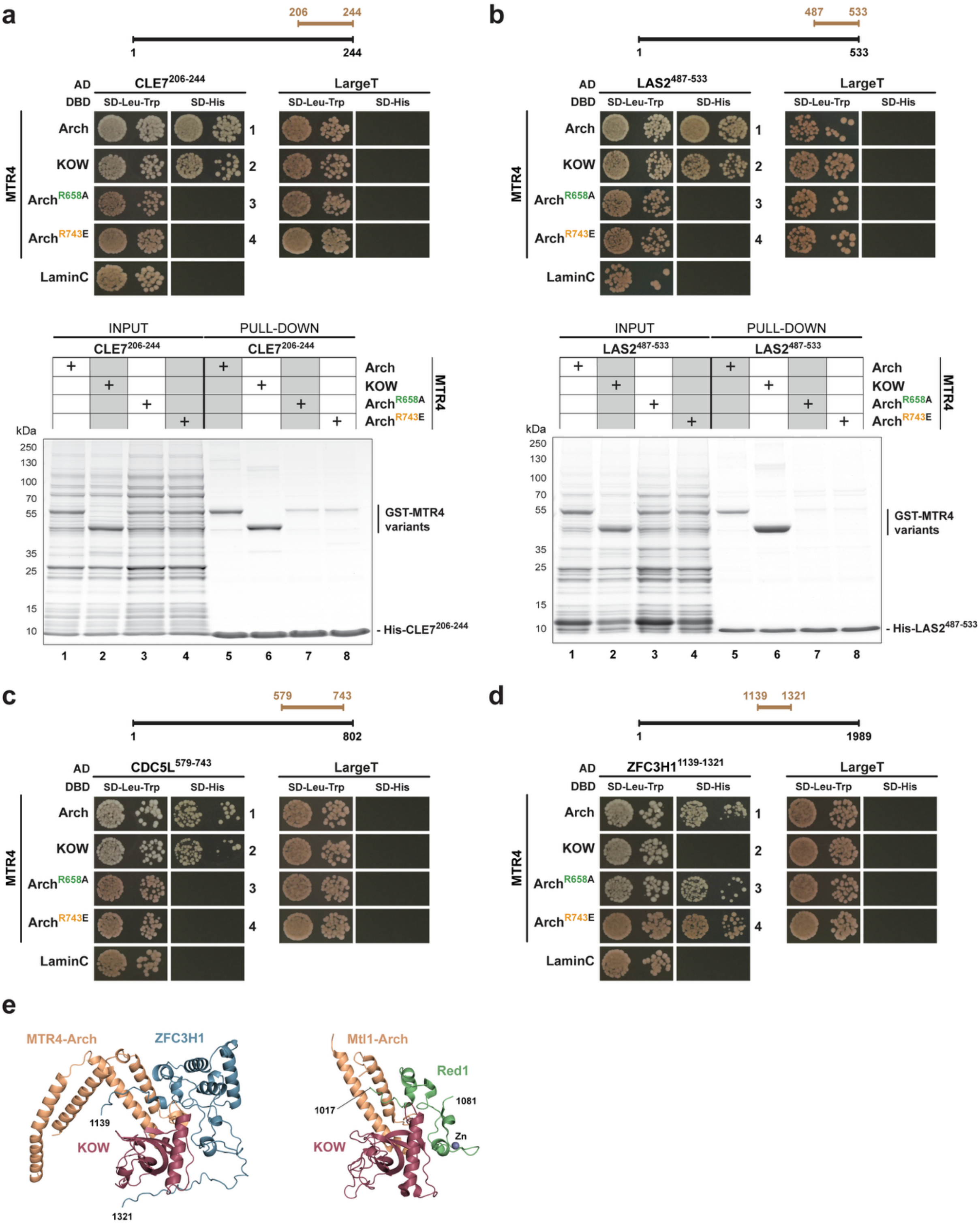

Supplementary Figure 2. MTR4-Arch interactors lacking a canonical AIM. Y2H analysis of MTR4-Arch interactions with **a** CLE7$^{206\text{-}244}$, **b** LAS2$^{487\text{-}533}$, **c** CDC5L$^{579\text{-}743}$, and **d** ZFC3H1$^{1139\text{-}1321}$. Plasmids encoding the tested proteins fused to the LexA DNA-binding domain (DBD) and GAL4 activation domain (AD) as indicated were transformed into the NMY32 strain. Transformants were spotted on selective SD-Leu-Trp and SD-His media and incubated at 30 °C for 3-4 days. LaminC and LargeT were used as

negative controls. (**a-b)** Lower panels: GST-tagged variants of MTR4 were co-expressed with His_6-$CLE7^{206-244}$ and His_6-$LAS2^{487-533}$ in *E. coli* cells and affinity-purified using a Ni-NTA resin. The bound proteins were analyzed by SDS-PAGE and visualized with Coomassie Blue staining. **e** AlphaFold2-derived model of the MTR4-Arch domain (residues 579-842) and $ZFC3H1^{1139-1321}$ fragment (left), and the crystal structure of *C. thermophilum* Mtl1-Arch:Red1 complex (PDB: 6YGU, right). AlphaFold2-predicted structures as PDB-format coordinate files are provided as Supplementary Data. Source data are provided as a Source Data file.

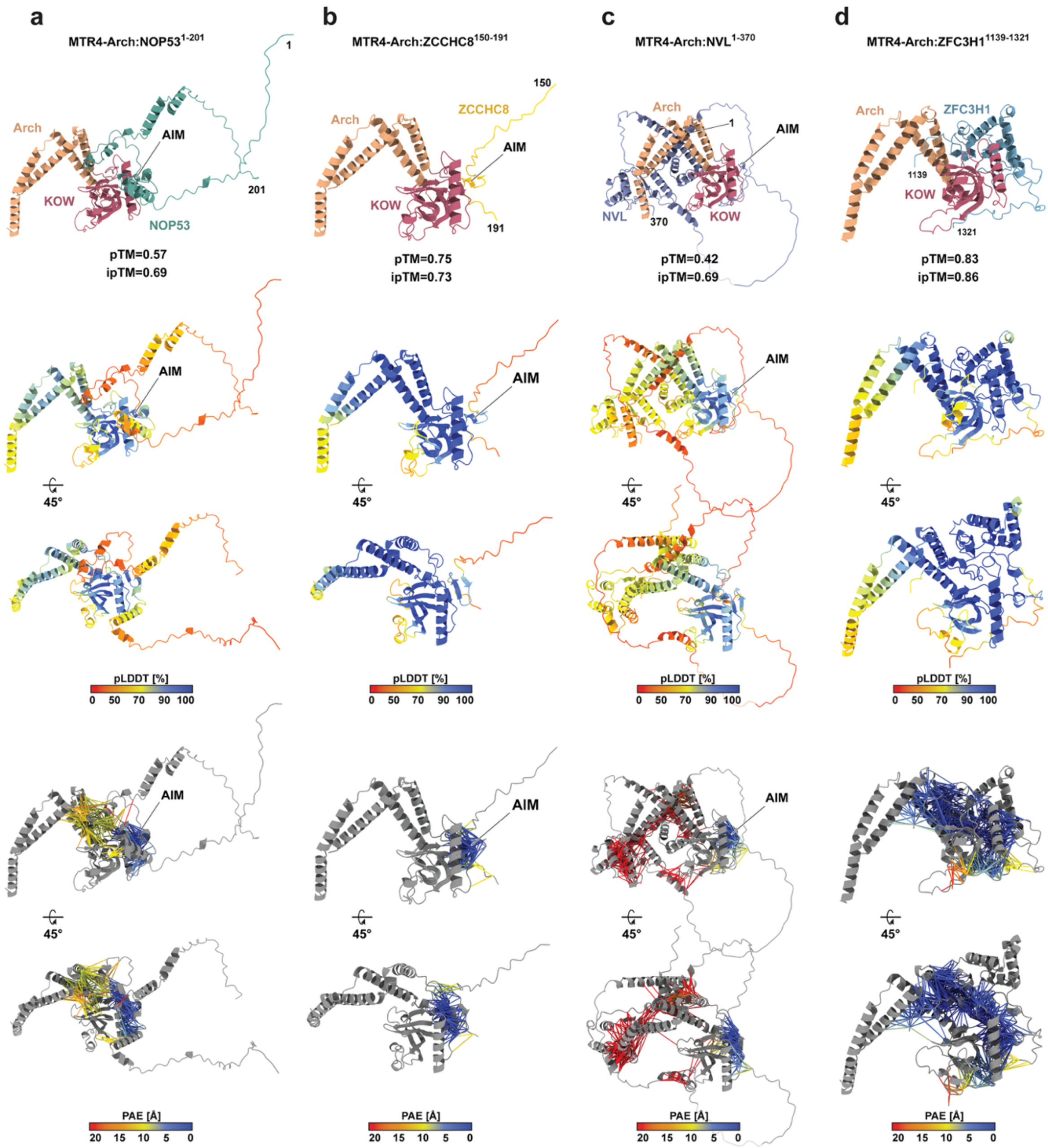

Supplementary Figure 3. AlphaFold2-derived models of MTR4-Arch domain (residues 579-842) in complex with the depicted NOP53, ZCCHC8, NVL or ZFC3H1 fragments. The prediction confidence was assessed using predicted local distance difference test (pLDDT), predicted aligned error (PAE) and pTM/ipTM scores. Pseudobonds reflecting the PAE between residue pairs were displayed for distances up to 8 Å. Structures were analyzed with ChimeraX (version 1.8)[1]. Models are shown for **a** MTR4-Arch:$NOP53^{1-201}$, **b** MTR4-Arch:$ZCCHC8^{150-191}$, **c** MTR4-Arch:NVL^{1-370}, and **d** MTR4-Arch:$ZFC3H1^{1139-1321}$. AlphaFold2-predicted structures as PDB-format coordinate files are provided as Supplementary Data.

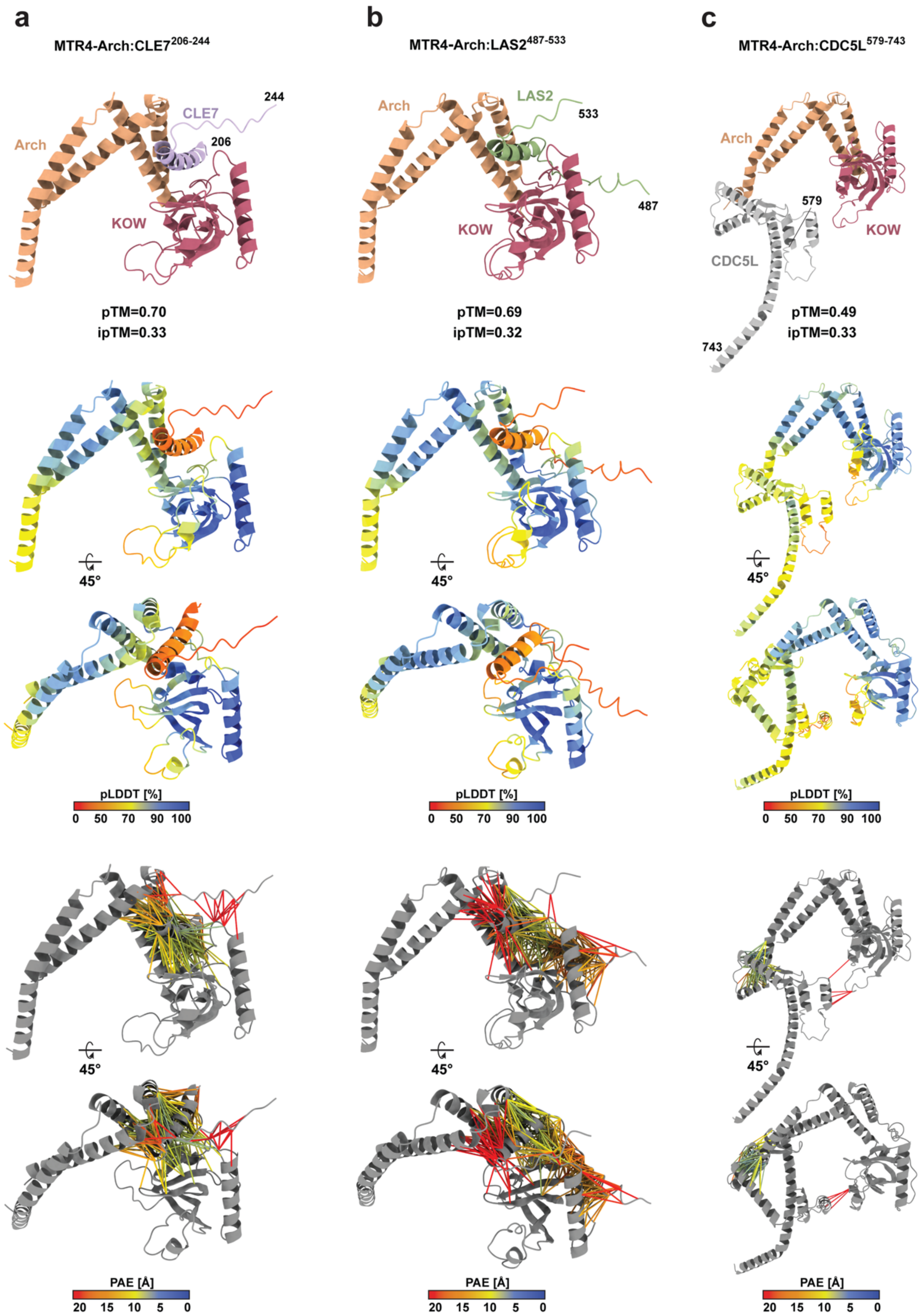

Supplementary Figure 4. AlphaFold2-derived models of MTR4-Arch domain (residues 579-842) in complex with the depicted CLE7, LAS2 or CDC5L fragments. The prediction confidence was assessed using predicted local distance difference test (pLDDT), predicted aligned error (PAE) and pTM/ipTM scores. Pseudobonds reflecting the PAE between residue pairs were displayed for distances up to 8 Å. Structures were analyzed with ChimeraX (version 1.8)[1]. Models are shown for **a** MTR4-Arch:CLE7$^{206\text{-}244}$, **b** MTR4-Arch:LAS2$^{487\text{-}533}$, and **c** MTR4-Arch:CDC5L$^{579\text{-}743}$. AlphaFold2-predicted structures as PDB-format coordinate files are provided as Supplementary Data.

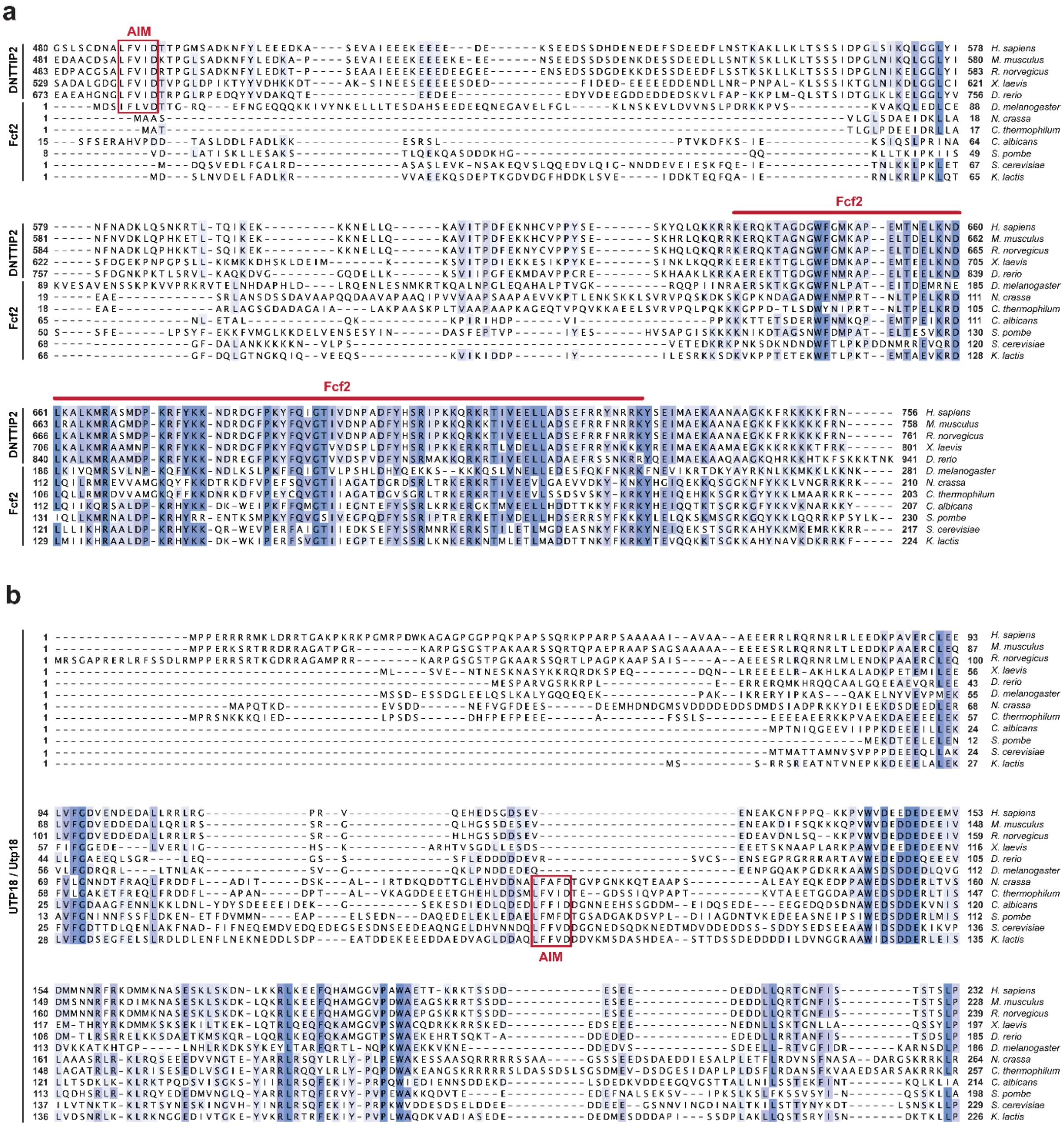

Supplementary Figure 5. Sequence alignments of DNTTIP2/Fcf2 and UTP18/Utp18 homologs among eukaryotes. Alignments were generated using ClustalO and visualized with Jalview[2]. Protein sequences were retrieved from the UniProt database[3]. **a** Sequence alignment of DNTTIP2/Fcf2 homologs. The conserved C-terminal region of these proteins corresponds to the Fcf2 domain (red line) and is preserved across both higher and lower eukaryotes. The N-terminal extension is present exclusively in higher eukaryotes and harbors a recognizable AIM sequence (boxed in red). **b** Sequence alignment of UTP18/Utp18 homologs. A recognizable AIM (boxed in red) is present in lower eukaryotes but absent from the homologs in higher eukaryotes.

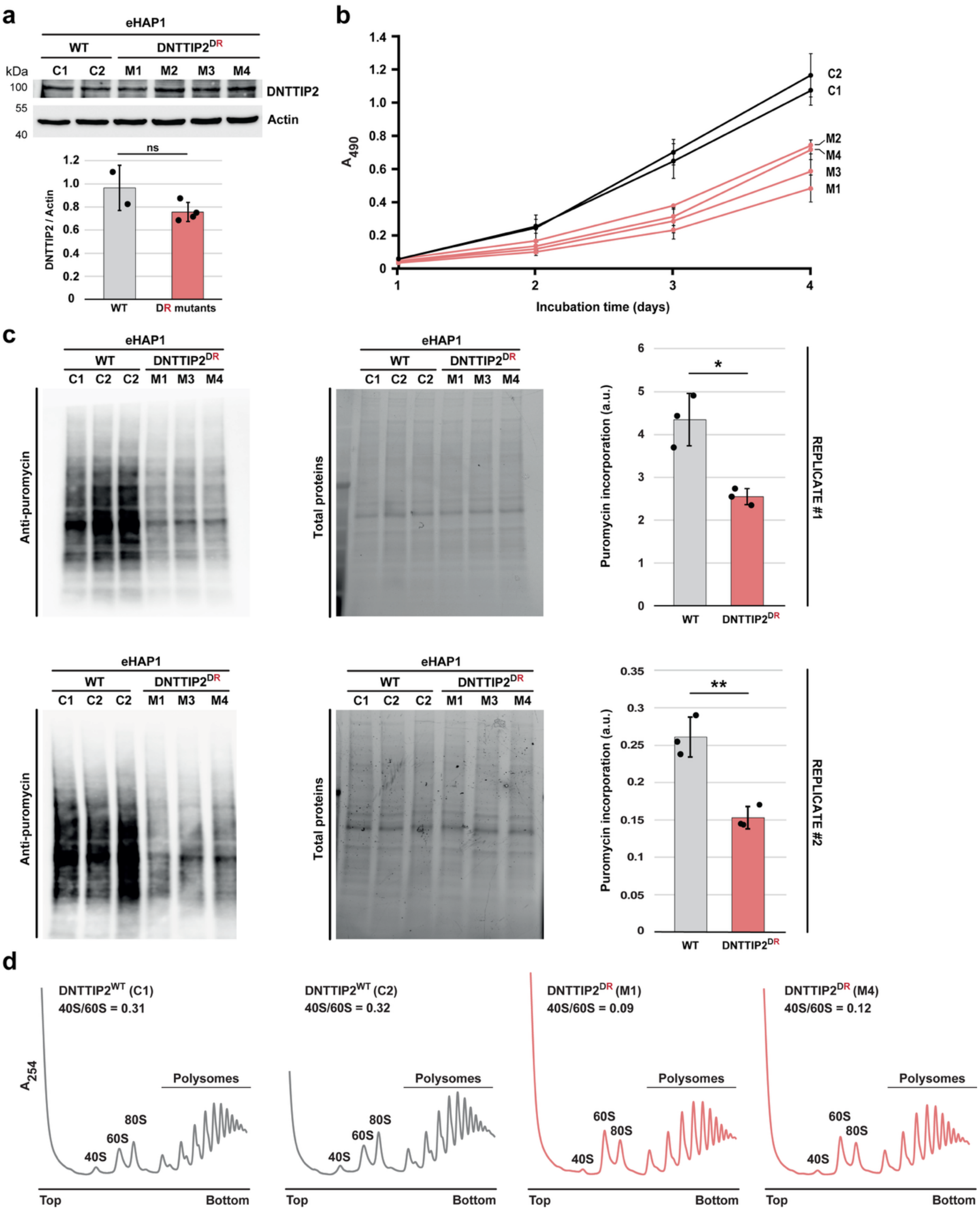

a
eHAP1
WT
DNTTIP2DR
kDa
C1 C2 M1 M2 M3 M4
100
55
40
DNTTIP2
Actin
ns
DNTTIP2 / Actin
WT
DR mutants
b
A490
C2
C1
M2
M4
M3
M1
Incubation time (days)
c
Anti-puromycin
Total proteins
Puromycin incorporation (a.u.)
REPLICATE #1
REPLICATE #2
d
DNTTIP2WT (C1)
40S/60S = 0.31
DNTTIP2WT (C2)
40S/60S = 0.32
DNTTIP2DR (M1)
40S/60S = 0.09
DNTTIP2DR (M4)
40S/60S = 0.12
A254
Polysomes
40S
60S
80S
Top
Bottom

Supplementary Figure 6. Integrity of $DNTTIP2^{AIM}$ is required for efficient cell proliferation and global protein synthesis. a Two control eHAP1 cell lines expressing wild-type DNTTIP2 (C1, C2) and four independent mutant cell lines expressing the $DNTTIP2^{DR}$ variant (M1-M4) were grown to mid-confluence. Total protein extracts from these cells were analyzed by western blotting using antibodies detecting DNTTIP2 or actin as a loading control. The signals were quantified using Image Lab software (Bio-Rad). The DNTTIP2 signals were normalized with respect to the actin signals. Data from two or four biological replicates, for respectively the wild-type (WT) and $DNTTIP2^{DR}$ mutant cell lines, are presented as mean (vertical columns) ± standard deviation (error bars). The individual data points are shown as black dots. Statistically significant differences were determined using a one-tailed Student's t-test (ns, not significant). **b** Proliferation of control (C1, C2) and mutant (M1-M4) eHAP1 cell lines assessed by MTS assay. The cell lines were seeded at about 1500 cells per well in 96-well plates, and MTS tetrazolium reagent was added after 1, 2, 3 and 4 days of growth followed by measurement of absorbance at 490 nm (A_{490}). The experiments were performed in five biological triplicates for the C1, C2, M1, M3 and M4 cell lines and one biological triplicate for the M2 cell line. **c** Global protein synthesis in control (C1, C2) and mutant (M1, M3, and M4) cell lines assessed by puromycin incorporation. Cells were treated with 1 µM puromycin for 1 hour, lysed in RIPA buffer, and equal amounts of total proteins were resolved on stain-free gels. Total protein fluorescence (central panels), anti-puromycin western blots (left panels), and quantification normalized to total proteins in each lane (right panels) are shown. Data represent means of three biological replicates (columns) ± standard deviation (error bars). The individual data points are shown as black dots. Statistically significant differences were determined using a one-tailed Student's t-test (*$p < 0.01$; **$p < 0.001$). The experiment was performed in duplicates. **d** Polysome profiling of control (C1, C2) and mutant (M1, M4) cell lines. Mid-confluent, cycloheximide-treated cultures were lysed, and cytoplasmic extracts were sedimented through 10%-50% sucrose gradients. The gradients were fractioned from top (low density) to bottom (high density), while continuously recording absorbance at 254 nm (A_{254}). The 40S/60S ratios were determined and depicted by calculating the relative heights of the 40S and 60S peaks according to the A_{254} traces using Excel software. Source data are provided as a Source Data file.

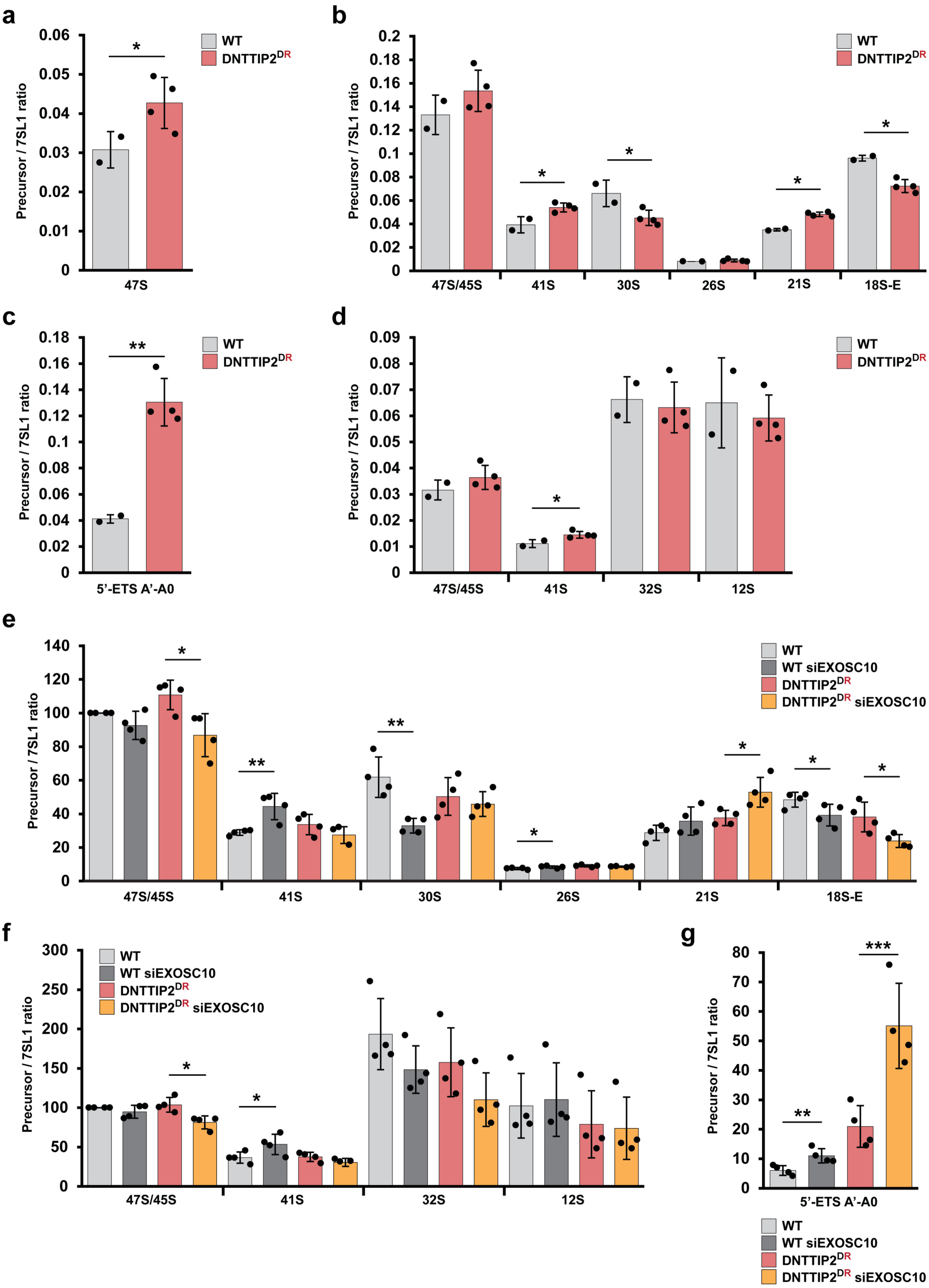
a
b
c
d
e
f
g
Precursor / 7SL1 ratio
WT
DNTTIP2DR
WT siEXOSC10
DNTTIP2DR siEXOSC10
47S
47S/45S
41S
30S
26S
21S
18S-E
32S
12S
5'-ETS A'-A0
*
**
***

Supplementary Figure 7. Quantification of the Northern blotting data. (a-d) Quantification of rRNA precursor levels in the Northern blotting experiments presented in main Fig. 3. Signal intensities for all rRNA precursors (Fig. 3c-g) and the 7SL1 RNA used as a loading control (Fig. 3b) were quantified using Multi Gauge software (FUGIFILM) and rRNA precursor signals were normalized with respect to the 7SL1 signal. **a** Normalized 47S precursor signal detected with probe 5'ETS #1 in Fig. 3c. **b** Normalized 47S/45S, 41S, 30S, 26S, 21S and 18S-E precursor signals detected with probe ITS1 #1 in Fig. 3d. **c** Normalized 5'ETS A'-A_0 fragment signal detected with probe 5'ETS #2 in Fig. 3f. **d** Normalized 47S/45S, 41S, 32S, and 12S precursor signals detected with probe ITS2 #3 in Fig. 3e. Data from two or four biological replicates, for respectively the control and DNTTIP2^{DR} mutant cell lines, are presented as mean (vertical columns) ± standard deviation (error bars). The individual data points are shown as black dots. Statistically significant differences were determined using bilateral Student's t-test (*$p < 0.05$; **$p < 0.01$; ***$p < 0.001$). (**e-g)** Quantification of the rRNA precursor levels in the Northern blotting experiments presented in main Fig. 6. The signal intensities for all rRNA precursors (Fig. 6c-g) and the 7SL1 RNA used as a loading control (Fig. 6b) were quantified using Multi Gauge software (FUGIFILM) and rRNA precursor signals were normalized with respect to the 7SL1 signal. **e** Normalized 47S/45S, 41S, 30S, 26S, 21S and 18S-E precursor signals detected with probe ITS1 #1 in Fig. 6d. **f** Normalized 47S/45S, 41S, 32S and 12S precursor signals detected with probe ITS2 #3 in Fig. 6e. **g** Normalized 5'ETS A'-A_0 fragment signal detected with probe 5'ETS #2 in Fig. 6f. Data from four independent experiments (biological replicates) are presented as mean (vertical columns) ± standard deviation (error bars). The individual data points are shown as black dots. Statistically significant differences were determined using bilateral Student's t-test (*$p < 0.05$; **$p < 0.01$; ***$p < 0.001$). Source data are provided as a Source Data file.

Supplementary references

1. Pettersen, E. F. *et al.* UCSF ChimeraX: Structure visualization for researchers, educators, and developers. *Protein Sci* **30**, 70–82 (2021).
2. Waterhouse, A. M., Procter, J. B., Martin, D. M. A., Clamp, M. & Barton, G. J. Jalview Version 2 – a multiple sequence alignment editor and analysis workbench. *Bioinformatics* **25**, 1189–1191 (2009).
3. Consortium, T. U. *et al.* UniProt: the Universal Protein Knowledgebase in 2025. *Nucleic Acids Res* **53**, D609–D617 (2025).
