# Supplementary material for: DNTTIP2 coordinates RNA exosome activities to ensure fidelity of human ribosome assembly
Source: Nat Commun. 2026 Aug 7;17:9488. doi: 10.1038/s41467-026-76536-x (PMC13542088; doi:10.1038/s41467-026-76536-x)
Supplement: Supplementary file 4 [file 41467_2026_76536_MOESM2_ESM.pdf]

Description of Additional Supplementary Files

Supplementary Data 1
Alphafold2 predicted structure of the MTR4-Arch (residues 579-842) and NOP53 (residues 1-201) complex.

Supplementary Data 2
Alphafold2 predicted structure of the MTR4-Arch (residues 579-842) and ZCCHC8 (residues150-191) complex.

Supplementary Data 3
Alphafold2 predicted structure of the MTR4-Arch (residues 579-842) and NVL (1-370) complex.

Supplementary Data 4
Alphafold2 predicted structure of the MTR4-Arch (residues 579-842) and CLE7 (residues 206-244) complex.

Supplementary Data 5
Alphafold2 predicted structure of the MTR4-Arch (residues 579-842) and LAS2 (residues 487-533) complex.

Supplementary Data 6
Alphafold2 predicted structure of the MTR4-Arch (residues 579-842) and CDC5L (residues 579-743) complex.

Supplementary Data 7
Alphafold2 predicted structure of the MTR4-Arch (residues 579-842) and ZFC3H1 (residues 1139-13213) complex.

Supplementary Data 8
Alphafold2 predicted structure of the MTR4-Arch (residues 579-842) and DNTTIP2 (residues 477-520) complex.
